# Extracellular Vesicle-Mediated Local and Systemic Communication in Metabolic Diseases

**DOI:** 10.3390/ijms27188287

**Published:** 2026-09-17

**Authors:** Rong-Ping Fan, Li-Ping Gou, Jin-Fang Ma, Jing-Min Yang, Jack Isaac Wood, Rui-Ning Zhang, Xiang-Hui Fu, Nan-Wei Tong

**Affiliations:** 1 Department of Endocrinology and Metabolism, Center for Diabetes and Metabolism Research, West China Hospital, Sichuan University, Chengdu 610044, China; 2023324025199@stu.scu.edu.cn (R.-P.F.); gouliping2022@wchscu.cn (L.-P.G.); majinfang.scu@foxmail.com (J.-F.M.); zhangrn0717@163.com (R.-N.Z.); 2 Department of Thoracic Surgery West China Biomedical Big Data Center, West China Hospital, Sichuan University, Chengdu 610044, China; d202280869@hust.edu.cn; 3 Department of Psychiatry and Neurochemistry, Sahlgrenska Academy, University of Gothenburg, 43180 Mölndal, Sweden; jack.wood.16@ucl.ac.uk; 4 Department of Neuroscience, Physiology and Pharmacology, University College London, Gower Street, London WC1E 6BT, UK; 5 Department of Endocrinology and Metabolism, Department of Biotherapy, Center for Diabetes and Metabolism Research, State Key Laboratory of Biotherapy and Cancer Center, West China Hospital, Sichuan University, No. 37, Guoxue Road, Chengdu 610044, China

**Keywords:** extracellular vesicle, intercellular communication, inter-organ communication, metabolic disease

## Abstract

Extracellular vesicles (EVs) have emerged as key mediators of intercellular communication, shuttling bioactive proteins, lipids, and nucleic acids between organs to maintain metabolic homeostasis. In this review, we provide an overview of recent advances in EV-mediated local and systemic communication across four interconnected metabolic diseases: diabetes, obesity, metabolic dysfunction-associated steatotic liver disease (MASLD), and sarcopenia, with a focus on the mechanistic roles of EVs in disease onset, adaptation, and progression. We highlight how EVs from the pancreas, adipose tissue, liver, and skeletal muscle propagate metabolic dysfunction across organs, and identify some shared cargo signatures, including miR-27a, miR-155, miR-146a-5p, miR-690, CXCL10, RBP4, ceramides, adipokine composition, and mitochondrial components, which may represent common pathogenic mechanisms. Current EV-based therapeutic strategies are discussed, and future efforts should prioritize the standardization of EV isolation and characterization to improve reproducibility, thereby facilitating clinical translation.

## 1. Introduction

Metabolic diseases, including diabetes [1], obesity [2], metabolic dysfunction-associated steatotic liver disease (MASLD) [3], and their progressive later-stage complications, represent a global health crisis on an epidemic scale. These conditions are closely linked, sharing common underlying disease mechanisms such as dysfunctional inter-organ communication [4]. Traditionally, metabolic homeostasis was thought to be primarily regulated by classical hormones [5]. However, this view cannot fully explain the complex and coordinated intercellular and inter-organ communication that contributes to metabolic dysfunction. In recent years, extracellular vesicles (EVs) have emerged as important mediators of such communication [6,7], thereby providing a novel mechanistic framework for understanding metabolic disorders [8].

**Figure 1:**
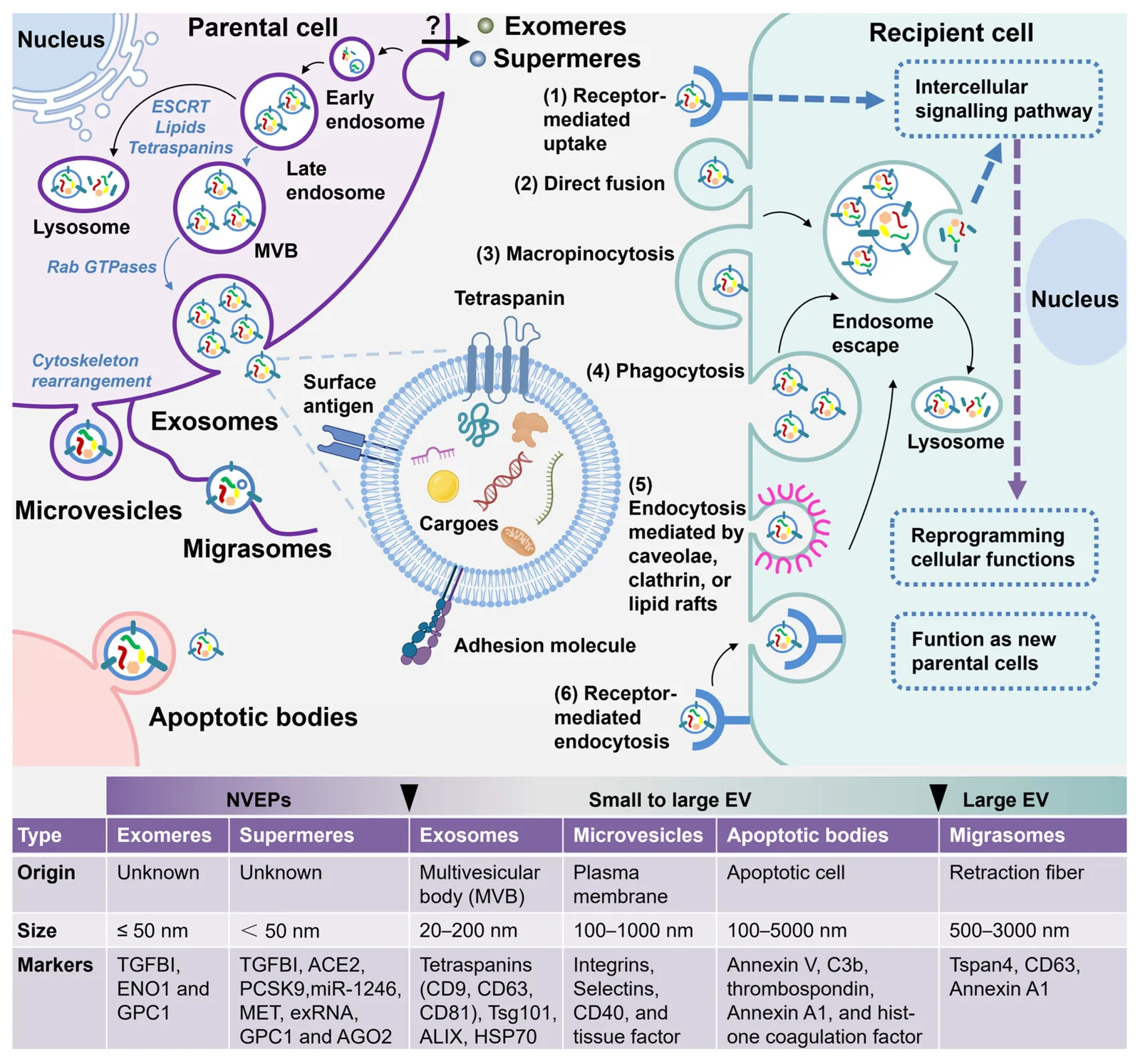
Biogenesis and characteristics of extracellular vesicles and non-vesicular extracellular nanoparticles. Extracellular vesicles (EVs) are lipid bilayer-enclosed particles released from cells through distinct biogenesis pathways. Microvesicles and apoptotic bodies are generated by the direct outward budding and fission of the plasma membrane. Exosomes originate as intraluminal vesicles within endosome-derived multivesicular bodies (MVBs); these MVBs subsequently fuse with the plasma membrane to release their vesicular contents into the extracellular space. Migrasomes are generated on the retraction fibers of migrating cells. In parallel, cells also release non-vesicular nanoparticles (NVEPs) lacking a lipid bilayer, such as exomeres and supermeres. EVs transport diverse molecular cargo, including proteins, lipids, nucleic acids, and even mitochondrial components, and can be taken up by the recipient cell via (1) receptor-mediated uptake, (2) direct fusion, (3) macropinocytosis, (4) phagocytosis, (5) endocytosis mediated by caveolae, clathrin, or lipid rafts, and (6) receptor-mediated endocytosis. Upon internalization, most EV cargo is sequestered within endosomes. From there, internalized EVs either fuse with lysosomes for degradation, or undergo endosomal escape to release functional cargo into the cytosol. This released cargo can activate intercellular signaling pathways and reprogram cellular functions. Furthermore, recipient cells may subsequently act as new parental cells and release their own EVs, thereby perpetuating or further amplifying the intercellular communication network. The biogenetic origin, typical size, and representative markers [18] of each extracellular particle type are presented in the schematic. Figures were created with BioRender (https://biorender.com/).

EVs are submicron-sized, lipid bilayer-enclosed particles secreted by virtually all cell types [7]. They are primarily classified into three major subtypes based on their biogenesis and size: exosomes, microvesicles, and apoptotic bodies [9] (Figure 1). Exosomes (20–200 nm) are formed within the endosomal system and released through the fusion of multivesicular bodies (MVBs) with the plasma membrane. Their biogenesis involves machinery such as the endosomal sorting complex required for transport (ESCRT), Rab GTPases, cytoskeletal proteins, and lipids such as ceramide [10]. In contrast, microvesicles (100–1000 nm; also referred to as ectosomes, microparticles, or shedding vesicles) bud directly from the plasma membrane, a process driven by cytoskeletal reorganization and the redistribution of lipids and proteins within the membrane [11]. Apoptotic bodies (1000–5000 nm) represent a subtype of microvesicles produced during cell death [12]. However, the EV family has expanded considerably over the past decade to include additional subtypes [13], such as migrasomes (500–3000 nm), which are large EVs released from retraction fibers during cell migration [14]. Non-vesicular extracellular particles (NVEPs) have also been described and include exomeres (≤50 nm) and supermeres (~25 nm), neither of which are enclosed by a lipid bilayer [15]. Currently available isolation techniques do not allow complete separation of these overlapping populations based solely on size or density [16]. Consequently, the minimal information for studies of extracellular vesicles (MISEV) 2023 guidelines [17] recommend classifying EVs into operational subgroups, such as small EVs (sEVs, <200 nm) and medium/large EVs (m/lEVs, >200 nm), based on the separation method employed, unless their biogenesis has been specifically investigated, given that the small EV population comprises both small ectosomes and exosomes. To avoid ambiguity, this review consistently uses the operational term “sEVs” to refer to membrane-enclosed vesicles unless specified otherwise.

EVs carry complex and bioactive molecular cargo, including nucleic acids (e.g., miRNAs, mRNAs, lncRNAs), proteins, lipids, mitochondrial components, and metabolites, which reflect the physiological or pathological state of their parental cell [19]. This cargo is delivered to target cells via diverse uptake mechanisms and activates intercellular signaling pathways upon internalization by recipient cells, thereby reprogramming cellular functions such as glucose uptake, insulin sensitivity, inflammatory responses, and tissue remodeling [20,21]. The established role of EVs in intercellular communication makes understanding their function in metabolic disease increasingly important.

While existing reviews have explored inter-organ crosstalk in metabolism, they either lack an EV-centric perspective [22,23,24,25] or focus solely on EVs derived from a single organ [26,27,28,29,30,31]; a comprehensive overview of multi-directional EV-mediated communication at both the local and systemic levels among key metabolic tissues (pancreas, adipose tissue, liver, skeletal muscle) is still lacking. This review summarizes the evidence on how EVs from these tissues mediate local microenvironmental changes in diabetes, obesity, MASLD/MASH, and sarcopenia, while also contributing to systemic pathological networks. Distinguishing these EV functions and mechanisms is essential for developing targeted therapeutic strategies. Furthermore, we compile EV cargoes reported across ≥2 disease models (Table 1), providing a cross-disease perspective that facilitates the rational selection of clinical targets with pleiotropic therapeutic potential.

## 2. Extracellular Vesicle-Mediated Local and Systemic Communication in Diabetes

### 2.1. EV-Mediated Local Communication in Diabetes

Type 1 diabetes mellitus (T1DM) is characterized by autoimmune-mediated destruction of pancreatic β-cells by autoreactive T cells, leading to reduced insulin secretion and loss of β-cell mass [59], whereas type 2 diabetes mellitus (T2DM) is characterized by non-autoimmune, heterogeneously progressive β-cell dysfunction and macrophage-mediated inflammation [60]. Emerging evidence suggests that EV-mediated crosstalk between immune cells and β-cells is closely associated with the pathogenesis of both T1DM and T2DM. Lymphocyte-derived sEVs carrying miR-142-3p, miR-142-5p, and miR-155 have been shown to promote pancreatic β-cell death and immune cell recruitment, potentially exacerbating β-cell apoptosis [61]. A similar pro-apoptotic effect was reported in T cell-derived EVs containing five tRNA-derived fragments [62]. Macrophage-derived EVs also affect β-cell function. miR-212-5p-carrying sEVs derived from M1-polarised macrophages may impair β-cell insulin secretion by targeting sirtuin 2 and suppressing the Akt/GSK-3β/β-catenin pathway [63].

β-cells may not merely be passive recipients of EVs but also active secretors, releasing EVs that may exert protective or harmful effects. For example, on the protective side, β-cells stimulated with proinflammatory interferons exhibited increased surface programmed death-ligand 1 (PD-L1) on their sEVs. This PD-L1 was then shown to bind to programmed death protein 1 (PD-1) on CD8+ T cells, inhibiting their proliferation and cytotoxicity and preventing β-cell destruction [64]. Additionally, stressed pancreatic β-cells were also shown to release EVs rich in damaged mitochondria, which were taken up by macrophages, subsequently triggering a Reg3g/exostosin-like glycosyltransferase 3-heparan sulfate-dependent signaling cascade that blocked NF-κB and downregulated purinergic receptor P2X7, thereby reprogramming macrophages toward an anti-inflammatory phenotype. This process, together with the subsequent mitophagic degradation of the damaged mitochondria, facilitated the clearance of sEVs from β-cells and ultimately restored islet tissue homeostasis [55].

On the harmful side, β-cell-derived EVs may impair neighboring β-cells under inflammatory [51] or lipotoxic stress [65]. β-cells exposed to proinflammatory cytokines produced sEVs carrying CXC motif chemokine ligand 10 (CXCL10), which bound to CXC motif chemokine receptor 3 (CXCR3) on neighboring β-cells, triggering inflammatory gene expression, recruiting T cells and macrophages, and ultimately forming a positive inflammatory feedback loop that aggravates β-cell dysfunction [47]. Proinflammatory cytokines also activated neutral sphingomyelinase 2, prompting β-cells to secrete ceramide-enriched sEVs that carried specific miRNAs to adjacent β-cells, where they disrupted insulin signaling and impaired glucose-stimulated insulin secretion (GSIS) [51]. Similarly, palmitate-induced lipotoxic stress stimulated β-cells to release sEVs that activated the TGF-BETA/SMAD3 pathway, thereby suppressing GSIS [65]. Interestingly, β-cell-derived sEVs containing miR-29 impaired insulin secretion by recruiting and activating monocytes/macrophages via TNF receptor-associated factor 3 (TRAF3) suppression [66]. Together, these findings suggest that stressed β-cells may affect neighboring cells through EV-mediated paracrine signaling and immune cell recruitment, although these interpretations are based largely on preclinical observations and require further validation.

Pancreatic stellate cells (PSCs)-derived EVs contribute to islet dysfunction related to T2DM and pancreatic fibrosis in pancreatogenic diabetes [67,68]. Under pathological stimuli, TGF-β1- and hypoxia-activated PSCs secreted sEVs carrying miR-140-3p and miR-143-3p [67] and miR-23a-3p [68], respectively, which promoted β-cell apoptosis. Conversely, pancreatic EVs from healthy patients suppressed human islet amyloid polypeptide formation, a pathology associated with T2DM [69], indicating a possible EV-mediated self-protective mechanism.

### 2.2. EV-Mediated Systemic Communication in Diabetes

#### 2.2.1. The Effects of AT-EVs on Pancreatic β-Cells

The effects of adipose tissue (AT)-derived EVs (AT-EVs) on pancreatic β-cells appear to be stage-dependent during the progression of T2DM. In the early stage of insulin resistance, β-cells exhibit a compensatory response by increasing insulin secretion and expanding β-cell mass to meet the increased insulin demand of peripheral organs [70]. For example, Ad-EVs isolated from obese and insulin-resistant mice enhanced first-phase GSIS in murine islets; mechanistically, the functional insulinotropic protein cargo in EVs transferred to pancreatic β-cells was found to augment insulinotropic G-protein-coupled receptor/cyclic AMP/protein kinase A signaling by increasing total protein abundance and phosphosite dynamics to enhance insulin secretion [71]. Similarly, small EVs derived from AT macrophages (ATM-sEVs) carrying miR-155 were able to suppress insulin secretion while promoting β-cell proliferation via Mafb inhibition, thereby supporting β-cell adaptation to obesity [37]. However, this compensatory regulation may be gradually replaced by the deleterious effects of increasingly inflamed Ad-EVs. These EVs carried pro-apoptotic signals such as miR-138-5p, which suppressed SOX4 and impaired the Wnt/β-catenin pathway, leading to β-cell apoptosis and impaired GSIS [72]. In contrast, sEVs originating from healthy adipocytes exerted opposing effects [73]. Blocking harmful AT-EVs may represent a promising therapeutic strategy for diabetes, as supported by evidence showing that inhibiting the delivery of miR-27a-5p-enriched small Ad-EVs to pancreatic islets improved glucose tolerance and enhanced insulin secretion in mice by upregulating the L-type calcium channel subtype CaV1.2 [74].

#### 2.2.2. The Role of Liver-Derived EVs in Diabetes

MASLD and T2DM frequently coexist, with MASLD severity correlating with an increased T2DM risk [75]. Emerging evidence suggests that liver-derived EVs may mechanistically link these conditions. While some hepatic sEVs may exert protective effects, with those carrying transmembrane proteins shown to enhance muscle glucose uptake and pancreatic insulin secretion in both healthy and MASLD mice [76], pathological liver-derived EVs may impair β-cell function. For instance, steatotic hepatocyte-derived small EVs (Hep-sEVs) containing miR-126a-3p promoted β-cell apoptosis by inhibiting insulin receptor substrate 2 [77]. Lipotoxic sEVs enriched with Fetuin-A induced islet inflammation by activating macrophage toll-like receptor 4 (TLR4), downregulating β-cell identity genes, and impairing GSIS [78]. Conversely, miR-7218-5p-deficient Hep-sEVs from obese mice promoted MIN6 β-cell proliferation via *Cd74* upregulation, suggesting a potential compensatory mechanism [79].

#### 2.2.3. The Role of Skeletal Muscle-Derived EVs in Diabetes

Skeletal muscle (SkM), as the principal site for insulin-mediated glucose disposal, may communicate with the pancreas via myokines and EVs [80,81]. SkM-sEVs carrying miR-16 from mice fed a high-palmitate diet promoted β-cell proliferation without affecting insulin secretion by reducing the expression of *Ptch1*, suggesting they may help expand β-cell mass during insulin resistance [82]. Furthermore, sEVs from muscle stem cells were shown to transfer regulatory signals to pancreatic β-cells, thereby activating autophagy through mTOR modulation and mitigating glucolipotoxic injury [83]. As the largest tissue involved in locomotion, SkM can respond to exercise by rapidly releasing sEVs into the circulation [84]. While their effects on AT and the liver are known, direct evidence of their impact on pancreatic islets requires future study.

**Figure 2:**
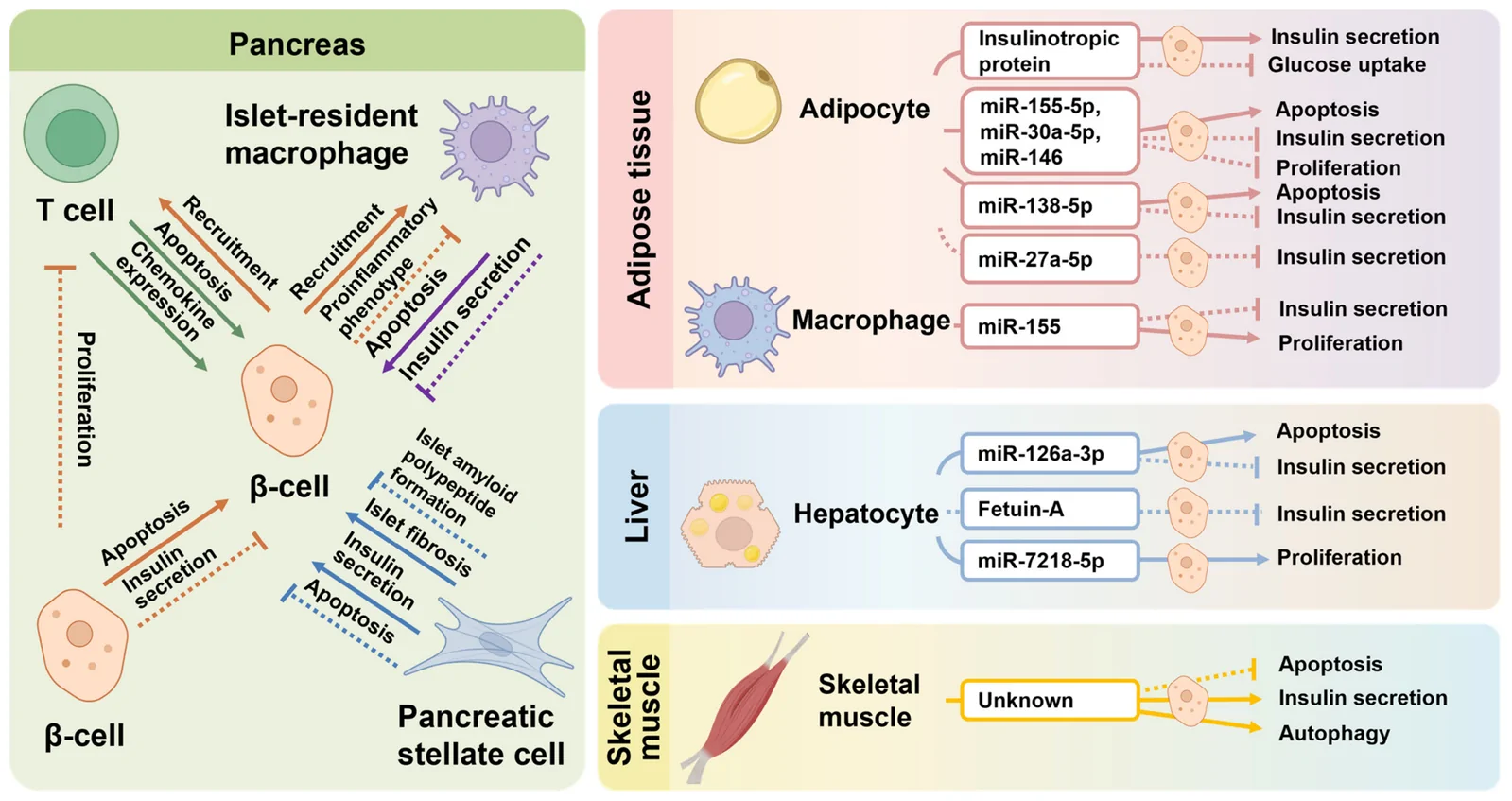
Extracellular vesicle-mediated local and systemic communication in diabetes. The (**left**) panel illustrates EV-mediated local communication in the diabetic microenvironment, highlighting contributions from T cells, islet-resident macrophages, β-cells, and pancreatic stellate cells. For example, T cell-derived EVs promote both β-cell apoptosis and chemokine expression in β-cells. The (**right**) panel depicts systemic communication, in which EVs derived from adipocytes, adipose tissue macrophages, hepatocytes, and skeletal muscle regulate β-cell fate and function. The central box outlines the corresponding EV cargo. The text following the arrow or bar indicates the EV-mediated effects on target cells or organs. For example, adipocyte-derived sEVs containing insulinotropic proteins promote insulin secretion but inhibit glucose uptake in β-cells. In the schematic, a solid arrow “→” indicates promotion, while a dashed bar “˧” denotes inhibition. The lines representing EV effects are color-coded according to the corresponding parental cells/tissues. Figures were created with BioRender (https://biorender.com/).

In summary, immune cell-mediated inflammatory responses within the local islet microenvironment play a critical role in driving pancreatic β-cell apoptosis; however, β-cells are not passive bystanders of their own destruction but instead display compensatory responses to mitigate damage from local or systemic stress signals. Figure 2 depicts the local and systemic mechanisms underlying EV-mediated communication in diabetes. Table 2 lists EV cargo and their corresponding parental cells, recipient cells, and mechanisms.

## 3. EV-Mediated Local and Systemic Communication in Adipose Tissue Remodeling and Obesity

### 3.1. EV-Mediated Local Communication in Adipose Tissue Remodeling

AT is a highly heterogeneous and dynamic organ composed of adipocytes and their stromal vascular fraction (SVF) cells, such as macrophages, endothelial cells (ECs), and adipose-derived stem cells (ADSCs). Generally, AT can expand in two ways: hypertrophy (the enlargement of existing adipocytes) and hyperplasia (the formation of new adipocytes from precursors known as preadipocytes). The differentiation of adipose progenitor cells into mature adipocytes is a tightly regulated process, termed adipogenesis [85]. In healthy states, these processes coexist and maintain a dynamic balance, with adipogenesis supporting the generation of small, metabolically active cells that enhance insulin sensitivity and limit ectopic lipid storage [86]. Conversely, in obesity, adipogenic capacity declines, shifting the balance toward hypertrophy. Hypertrophic adipocytes experience hypoxia and mechanical stress due to increased contact with neighboring cells and extracellular matrix components, which trigger inflammation, fibrosis, and adipokine dysregulation [86]. EVs mediate paracrine signaling between adipocytes and neighboring cells. EVs isolated from whole AT (AT-EVs) reflect mixed cellular origins, while Ad-EVs specifically originate from adipocytes.

AT-EVs play pleiotropic roles in adipogenesis, angiogenesis, and macrophage regulation. miR-450a-5p and miR-122 carried by AT-sEVs promoted adipogenesis in ADSCs [34,87]. AT-sEVs also enhanced angiogenesis [88], promoted M1-to-M2 macrophage polarization [89], and increased macrophage infiltration and recruitment of adipocyte precursors and ECs, supporting their potential role in AT regeneration and tissue repair [90]. However, the specific cargo mediating these effects was not characterized in these studies.

Importantly, the molecular composition of EVs dynamically reflects cellular differentiation states [91]; during 3T3-L1 adipogenesis, EV-associated preadipocyte factor-1 levels decrease while adiponectin levels increase, reflecting the transition from preadipocytes to mature adipocytes [91]. This adipogenic information can be transferred between cells, as EVs derived from human adipose-derived stem cells (HASCs) undergoing white or beige adipogenic differentiation promoted the corresponding white or beige differentiation in recipient HASCs via adipogenesis-related adipokines, including adiponectin, adipsin, angiopoietin-like 4, and insulin-like growth factor-1 [53]. Adiponectin is mainly expressed in adipocytes and has been used as a marker of Ad-EVs [28]. Functionally, adiponectin-positive AT-EVs induced a more pronounced AT macrophage (ATM) phenotype in monocytes compared to adiponectin-negative EVs [92], highlighting adipocyte-macrophage crosstalk mediated by distinct EV subpopulations [23].

Multiple studies have demonstrated that AT-EVs from metabolic disease models can promote macrophage M1 polarization and insulin resistance through diverse molecular cargo, including retinal binding protein 4 (RBP4) [49], Sonic Hedgehog [93], miR-155 [38] and EVs lacking miR-145-5p [94]. In addition, sEVs secreted by mature adipocytes containing miR-34a can be transported into macrophages, thereby suppressing the polarization of anti-inflammatory M2 macrophages by downregulating Kruppel-like factor 4 [95]. However, some Ad-sEVs carrying beneficial cargo may promote macrophage polarization toward an M2 phenotype, thereby ameliorating adipocyte inflammation. Examples of such beneficial cargo include α-ketoglutarate induced by melatonin [96] and active signal transducer and activator of transcription 3 carried by ADSC-derived EVs [97].

In addition to modulating the macrophage phenotype, Ad-EVs participate in regulating lipid accumulation and lipogenesis. Lipid-filled EVs released by adipocytes can be taken up by ATMs, thereby serving as a source of lipids for local macrophages [98]. Moreover, the daily release rate of such lipid-filled sEVs was more than two-fold higher in obese mice than in lean mice, which might be a mechanism of lipid release from adipocytes independent of canonical lipolysis [98]. Similarly, large adipocytes released EVs harboring glycosylphosphatidylinositol-anchored proteins, Gce1 and CD73, which transferred lipid droplet- and adipokine-specific transcripts, including four types of microRNAs, into small adipocytes, promoting lipogenesis and adipocyte hypertrophy [99]. The above evidence indicates that EVs may play a crucial role in the mechanism of intercellular lipid transfer.

EVs also mediated communication from SVF cells to adipocytes. miR-210 in sEVs derived from RAW264.7 macrophages cultured under high glucose conditions suppressed glucose uptake and mitochondrial function in adipocytes by inhibiting NADH oxidoreductase subunit A4 expression [100]. Similarly, ATM-derived sEVs loaded with miR-210-3p reduced glucose uptake in adipocytes and promoted insulin resistance in adipocytes, skeletal muscle cells, and hepatocytes by inhibiting GLUT4 expression [101]. In addition, adjacent ECs can deliver caveolin-1 (Cav1)-containing sEVs to adipocytes, thereby sustaining detectable Cav1 levels even after Cav1 gene ablation [102]. This evidence offers new insights into the EV-mediated transfer event existing between adipocytes and SVF cells.

Brown adipose tissue (BAT) also secretes EVs that mediate diverse forms of local cell-to-cell communication. Under obese conditions, brown adipocytes release EVs containing damaged lipids and mitochondria, which are taken up by lipid-associated macrophages via CD36, inducing a foam cell-like phenotype and increased secretion of TGF-β1. TGF-β1 subsequently suppresses brown adipocyte-specific gene expression in BAT by inducing aldehyde dehydrogenase 1 family member A1 [56]. Thermogenically stressed brown adipocytes also produce sEVs containing mitochondrial fragments; re-uptake of these sEVs by brown adipocytes reduces PPARγ and UCP1 expression, impairing thermogenesis, whereas macrophages phagocytose and clear these EVs, preventing their accumulation and preserving BAT function [103]. Conversely, BAT-derived sEVs carrying specific lncRNAs (e.g., AK029592) are taken up by adipose stem cells and preadipocytes, inducing beige adipocyte differentiation [104]. Moreover, enhanced release of sEVs from mitochondrial transcription factor A-overexpressing brown adipocytes enhances oxidative phosphorylation and mitochondrial activity in adjacent brown adipocytes, thereby conferring resistance to obesity [105]. In contrast, white adipocyte-derived sEVs containing miR-23b target E74-like ETS Transcription Factor 4 in BAT, reducing glucagon-like peptide-1 receptor and mitochondrial biogenesis, ultimately inhibiting thermogenesis and promoting obesity [106]. Thus, BAT-EVs mediate intercellular crosstalk, with their net effects on thermogenesis and obesity reflecting a dynamic balance between deleterious signals from stressed/obese BAT and AT, and beneficial signals from healthy or genetically modified brown adipocytes.

### 3.2. EV-Mediated Systemic Communication in Adipose Tissue Remodeling and Obesity

#### 3.2.1. The Effects of Pancreatic β-Cell-Derived EVs on Adipose Tissue Remodeling

Our previous study suggested that miR-26a from β-cell-derived sEVs, once taken up by adipocytes, was found to reduce adipocyte hypertrophy and insulin resistance [107]. However, more investigation is required to study how pancreatic islet-derived EVs affect adipocytes.

#### 3.2.2. The Effects of Liver-Derived EVs on Adipose Tissue Remodeling and Obesity

The effects of liver-derived EVs on adipocytes depend on the type of inducing factors and the stage of exposure. Although liver-derived sEVs from both high-fat diet (HFD) and high-fat/sucrose diet can be taken up by adipocytes and promote lipid accumulation, they exhibit different influences on AT lipid metabolism [108]; EVs induced by short-term HFD feeding tend to facilitate adipogenesis, while those induced by long-term HFD tend to drive lipogenesis. Hep-sEVs induced by long-term lipid overload and containing Let-7e-5p affected adipose remodelling by targeting *Pgc1α*; the secretion of these Hep-sEVs was regulated by peroxisome proliferator-activated receptor gamma-mediated geranylgeranylation of Rab27A [35]. Sometimes, the stage-specific dynamic changes in Hep-sEV cargo with different exposure durations can even produce opposing effects. In early obesity, hepatocyte sEVs containing miR-3075 were transferred to AT, SkM, and the liver and exerted insulin-sensitizing effects by inhibiting FA2H. In contrast, in chronic obesity, miR-434-3p rather than miR-3075 was highly expressed in Hep-sEVs and indirectly promoted insulin resistance by inducing obesity-related tissue inflammation [109]. Interestingly, certain overexpressed molecules in the liver can improve AT metabolism via EVs. For example, Hep-sEVs enriched with miR-130a-3p have been shown to alleviate insulin resistance, reduce lipogenesis, and promote glucose uptake by inhibiting pleckstrin homology domain leucine-rich repeat protein phosphatase 2 in adipocytes [110]. Similarly, transmembrane 4 L6 family member 5 (TM4SF5)-modified Hep-sEVs targeted BAT to facilitate glucose clearance and thermogenesis through an mTOR pathway-dependent but UCP1-independent pathway [111], exerting an insulin-like function that alleviates hyperglycemia [111]. Extending this regulatory paradigm to environmental stimuli, such as cold exposure, can also reshape Hep-EV function. Specifically, cold exposure triggered hepatic secretion of miR-293-5p-enriched sEVs, which were taken up by BAT. The delivered miR-293-5p suppressed Ten-eleven translocation methylcytosine dioxygenase 1 (TET1) expression and induced transcriptional reprogramming, leading to enhanced thermogenic activation. This EV-mediated liver-BAT communication improved systemic metabolic homeostasis, as evidenced by the attenuation of diet-induced obesity and metabolic dysfunction in vivo following miR-293-5p agonism treatment [112].

#### 3.2.3. The Impacts of SkM-Derived EVs on Adipose Tissue Remodeling and Obesity

In obesity, SkM-derived EVs may serve as mediators of metabolic deterioration, with altered cargo profiles driving lipotoxic accumulation in adipocytes and muscle atrophy [113]. Conversely, the majority of exercise-induced SkM-EVs released into the circulation exert beneficial metabolic effects [114]. Aerobic exercise stimulated the production of EVs enriched with proteins associated with mitochondrial biogenesis and fatty acid β-oxidation, which was able to be endocytosed by metabolic organs, especially the liver and AT, improving glucose tolerance, reducing lipid accumulation, and alleviating liver damage and atherosclerosis in obese mice [58]. Similarly, resistance exercise-induced SkM-sEVs containing muscle-specific miR-1 were preferentially taken up by epididymal white adipose tissue (eWAT) and exerted lipolytic effects, and such beneficial regulatory functions have been validated in both preclinical models and human studies [115,116]. The muscle-fat interaction is dynamic, as sEVs from proliferating versus differentiating C2C12 myocytes exert opposing effects. Proliferation-stage sEVs enriched with miR-146a-5p were found to suppress differentiation and adipogenesis, whereas sEVs from differentiating C2C12 myocytes exerted the opposite effects. The former also reversed the obese phenotype in SkM-specific miR-146a-5p knockout mice [42].

**Figure 3:**
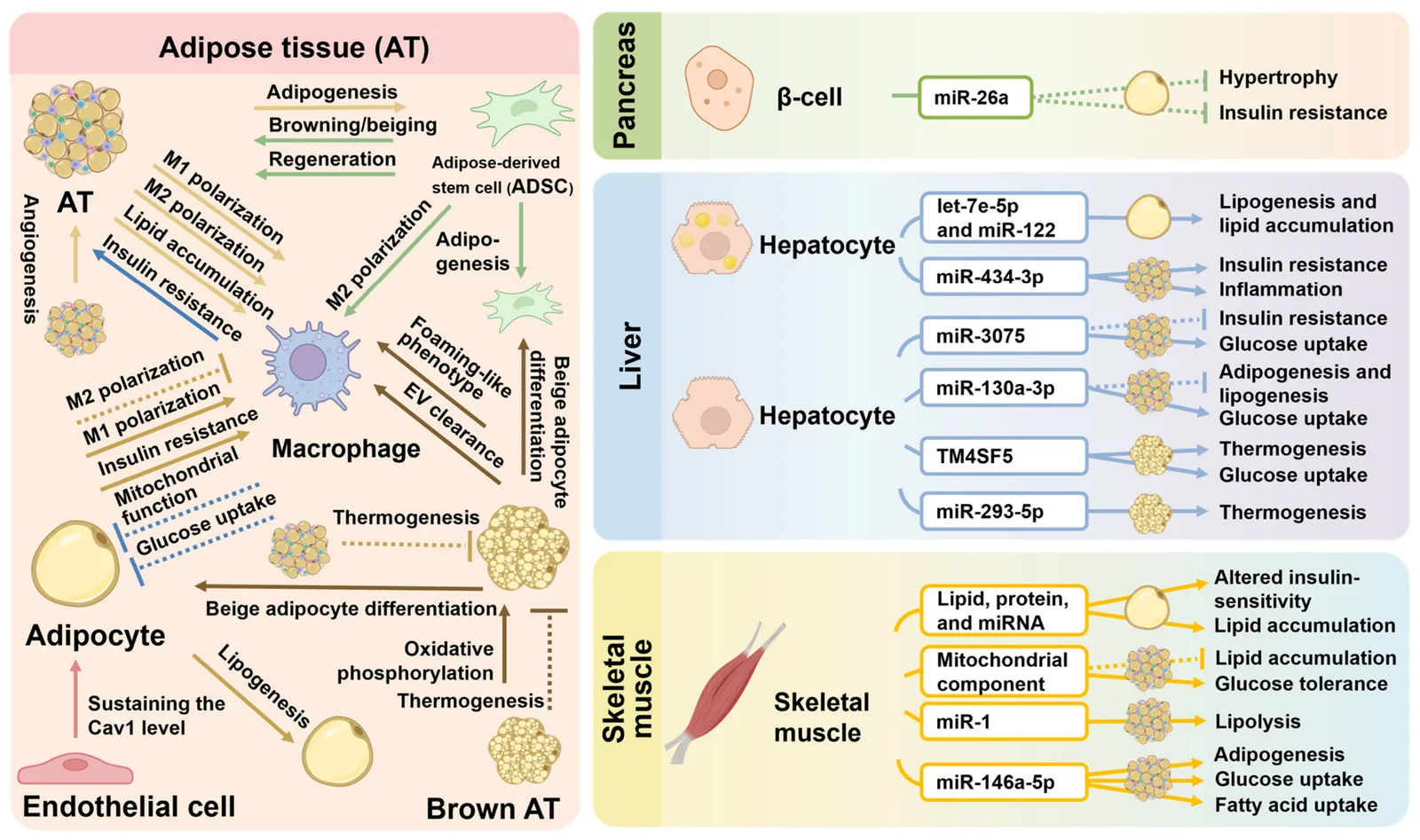
Extracellular vesicle-mediated local and systemic communication in adipose tissue remodeling and obesity. The (**left**) panel details the local EV-mediated crosstalk within adipose tissue, involving whole adipose tissue, adipocytes, adipose tissue macrophages, adipose-derived stem cells, and adipose endothelial cells. For example, adipocyte tissue-derived EVs are involved in promoting lipid accumulation and M1/M2 polarization in macrophages. The (**right**) panel summarizes the systemic EV signals originating from pancreatic β-cells, hepatocytes (including lipotoxic hepatocytes), and skeletal muscle, which modulate adipose tissue remodeling and metabolism. The central box outlines the corresponding EV cargo. The text following the arrow or bar indicates the EV-mediated effects on target cells or organs. For example, pancreatic β-cell-derived EVs containing miR-26a were able to inhibit adipocyte hypertrophy and insulin resistance. In this schematic, a solid arrow “→” indicates promotion, whereas a dashed “˧” represents inhibition. The lines representing EV effects are color-coded according to the corresponding parental cells/tissues. Figures were created with BioRender (https://biorender.com/).

Taken together, locally and systemically derived EVs converge on AT to drive its remodeling via multifaceted regulatory actions, targeting not only angiogenesis, adipogenesis and mitochondrial function, but also macrophage polarization, metabolic reprogramming of glucose and lipids, and the regulation of insulin sensitivity. Figure 3 illustrates the local and systemic mechanisms of AT remodeling and obesity-associated EVs. Table 3 provides EV cargoes and their corresponding parental cells, recipient cells, and mechanisms.

## 4. EV-Mediated Local and Systemic Communication in MASLD and MASH

### 4.1. EV-Mediated Local Communication in the Liver Microenvironment and Pathology of MASLD and MASH

As a pleiotropic endocrine organ, the liver absorbs nutrients from the hepatic sinusoid postprandially, making it one of the first organs to access absorbed nutrients and enabling it to influence metabolism in local and distal organs [35]. Part of the liver’s role in homeostatic regulation is achieved through EVs [76,117], which contribute to the progression of MASLD in response to elevated levels of fatty acids resulting from high-calorie diets and sedentary behavior [118]. MASLD is characterized by liver steatosis, inflammation, and fibrosis [119]. These pathological processes are closely associated with the interaction between hepatocytes and surrounding cells, such as hepatic stellate cells (HSCs, the primary source of extracellular matrix in the liver) [120], hepatic macrophages (HMs), and liver endothelial cells.

Lipotoxic hepatocytes release EVs that contribute to the activation of HSCs in MASLD and MASH [121,122,123]. The cargo of these pro-fibrotic Hep-EVs is diverse and includes molecules such as miR-128-3p [123] and unknown cargo [121,122], as well as additional mediators summarized in the review by Eguchi A et al. [27]. Among these, two well-characterized cargo components have been highlighted: iron [124] and mannan-binding lectin serine protease 1 (MASP1) [125]. Specifically, iron-loaded EVs were aberrantly secreted from hepatocytes and taken up by HSCs, leading to iron overload and reactive oxygen species (ROS)-mediated fibrogenic activation [124]. Similarly, upregulation of β-arrestin 1 (ARRB1) in stressed hepatocytes promoted the release of sEVs enriched in MASP1, which led to HSC activation via the p38 mitogen-activated protein kinase (MAPK)/activating transcription factor 2 (ATF2) signaling pathway, thereby driving liver fibrogenesis [125]. Beyond these, we have incorporated lipotoxic Hep-EV-associated cargo not previously reviewed, including miR-1297 [126], miR-27a [33], LIM Domain and Actin Binding 1 (LIMA1) [127], fatty acid synthase (FASN) [128], miR-107 [129], and Pumilio 1 (PUM1) [130]. miR-1297 modulated the PTEN/PI3K/AKT pathway to facilitate HSC activation and proliferation [126]. miR-27a and LIMA1 inhibited PINK1-dependent mitophagy, thereby inducing mitochondrial dysfunction and HSC fibrogenesis [33,127]. FASN exacerbated oxidative stress in HSCs [128]. Interestingly, miR-107-containing sEVs promoted HSC activation via both direct and indirect pathways. Directly, miR-107-containing sEVs activated Wnt signaling by suppressing dickkopf-1 (DKK1) to accelerate HSC proliferation and activation. Indirectly, it inhibited Foxp1 in CD4+ T cells to induce Th9 cell differentiation and IL-9 release, which further activated the Raf/MEK/ERK cascade in HSCs and aggravated HSC activation [129]. In contrast, sEVs with downregulated PUM1 failed to suppress the PUM1 target *Tropomyosin 4* mRNA, ultimately contributing to aberrant HSC activation [130]. Furthermore, steatotic Hep-EVs with unknown cargo promoted senescence in HSCs [131].

Lipotoxic Hep-EVs also promoted monocyte recruitment and/or HM activation. Several associated cargo molecules have been reviewed by Eguchi A et al., including CXCL10 [48], tumor necrosis factor-related apoptosis-inducing ligand (TRAIL) [132], and C16:0 ceramide [52]. These cargo molecules affect macrophages through different receptors or mechanisms, leading to upregulated expression of pro-inflammatory cytokines, which exacerbate liver injury and inflammation. Activation of mixed lineage kinase 3 (MLK3) is essential for the release of CXCL10-enriched sEVs [48]. Activation of the death receptor 5 (DR5)/Rho-associated coiled-coil containing protein kinase 1 (ROCK1) pathway is critical for the release of TRAIL-containing sEVs following lipid stimulation [132]. The IRE1α/X-box binding protein 1 (XBP1) signaling axis promoted the transcription of serine palmitoyltransferase, increasing ceramide biosynthesis, which in turn drove the release of ceramide-laden sEVs [52].

Additional lipotoxic Hep-EVs implicated in macrophage activation and/or monocyte recruitment carry cargo such as miR-192-5p [133], miR-9-5p [134], miR-122 [36], sphingosine 1-phosphate (S1P) [135], and active integrin β1 [136]. Among these, miR-192-5p and miR-9-5p promoted M1 macrophage polarization by suppressing the phosphorylation of AKT and Forkhead box transcription factor O1 (FOXO1) [133] or the expression of transglutaminase 2 (TGM2) in macrophages [134]. Moreover, miR-122 facilitated EV uptake by recipient macrophages and subsequent hepatic inflammation [36]; S1P promoted macrophage chemotaxis via S1P receptor 1 on macrophages [135], and active integrin β1 enhanced monocyte adhesion to liver sinusoidal endothelial cells (LSECs), a critical step for hepatic infiltration and differentiation into pro-inflammatory macrophages [136].

In addition to monocyte-derived macrophages, Hep-EVs may also polarize Kupffer cells (KCs), the liver’s resident macrophages, toward a pro-inflammatory phenotype, thereby worsening liver pathology. Specifically, RBP4-carrying Hep-sEVs promoted M1-like KC polarization, driving pro-inflammatory cytokine production and lipid accumulation in hepatocytes, thereby accelerating MASLD progression [50]. Similarly, Hep-sEVs containing miR-122-5p induced M1 polarization of KCs in liver ischemia–reperfusion injury [137]. In addition, lipotoxic Hep-sEVs delivered saturated fatty acids (SFAs) to macrophages, including KCs, triggering hepatic inflammation and contributing to hepatocyte insulin resistance [138].

Furthermore, lipotoxic Hep-sEVs delivered O-linked N-acetylglucosamine glycosyltransferase (OGT) to LSECs, enhancing O-GlcNAcylation of transcription factor HNF1α. This promoted HNF1α nuclear translocation and Angiopoietin-2 (Ang-2) transcription, thereby driving LSEC capillarization and implicating the OGT-HNF1α-Ang-2 axis as a key mediator of vascular dysfunction in MASLD [139].

EVs from non-parenchymal cells can also modulate hepatocyte function. TGFβ1-activated HSCs released miR-99a-5p-containing sEVs, promoting hepatocyte apoptosis [140], while KC-derived sEVs delivered miR-690 to hepatocytes and HSCs, with KCs serving as a key source of miR-690. The delivered miR-690 modulated recipient cell functions by suppressing NAD+ kinase. Notably, miR-690 levels declined during MASH progression, and its restoration restored KC activity and attenuated HSC fibrogenesis, inflammation in monocyte-derived hepatic macrophages, and hepatocyte lipogenesis [45].

Beyond hepatocyte-directed effects, EVs enable crosstalk between non-parenchymal cells, forming feed-forward loops that amplify HSC activation. For example, HSC-derived sEVs shuttled connective tissue growth factor between HSCs, potentially enhancing profibrotic signaling [141]; macrophage-derived miR-103-3p-containing sEVs promoted HSC activation [142]; and LSEC-derived EVs lacking miR-342-5p reduced inhibitory signals to HSCs, thereby promoting their activation and accelerating liver fibrosis [143].

Together, these studies support the concept that EVs mediate intercellular communication that contributes to MASLD progression and suggest that targeting HSC and HM activation may represent a promising therapy. For instance, the natural compounds catalpol and salidroside have been shown to modulate EV activity and content: catalpol reduced Rac1-GTP levels [144], and salidroside increased miR-146a-5p in Hep-sEVs [145]. Both these compounds alleviated fibrosis by inhibiting HSC activation in CCl_4_-induced liver fibrosis models, although such models lack several key features of MASLD/MASH [146]. Further studies in more clinically relevant models are needed to validate the therapeutic potential of such interventions.

### 4.2. EV-Mediated Systemic Communication in MASLD and MASH

#### 4.2.1. The Effects of Pancreatic β-Cell-Derived EVs on the Hepatic Metabolism

Evidence on pancreas-derived EVs primarily focuses on pancreatic islet β-cell EVs, whose regulatory effects on insulin-responsive organs are modulated by metabolic stress. Under lipotoxic conditions, β-cell-derived EVs impaired hepatic insulin sensitivity [147,148]. sEVs containing miR-29s additionally increased hepatic glucose output without altering lipid metabolism [147]. Under glucotoxic conditions, β-cell-derived sEVs containing miR-375-3p suppressed hepatic glycogenesis by inhibiting the *Rbpj* gene and downstream AKT/GSK signaling in hepatocytes [149]. Conversely, protective islet-derived sEVs enriched in miR-223 enhanced GLUT4-mediated glucose uptake in SkM and liver [150]. Similarly, our previous research suggested that β-cell-specific miR-26a overexpression improved hepatic insulin sensitivity through the transfer of miR-26a-enriched sEVs [107]. Additionally, islet-derived sEVs from co-transplantation models modulated hepatocyte function in a dose-dependent manner [151]. These findings highlight the diverse and context-dependent roles of β-cell EVs in inter-organ metabolic communication.

#### 4.2.2. The Effects of AT-EVs on the Liver Metabolism and Pathology of MASLD and MASH

AT-EVs exert bidirectional effects on hepatic insulin sensitivity. While many AT-EVs promote insulin resistance in liver and/or muscle cells through various pathogenic miRNAs and adipokines [54,152,153,154,155] such as miR-222 [154] and miR-141-3p [155], modified AT-sEVs carrying human kallistatin [156] and M2 macrophage-derived sEVs carrying miR-690 [43,44] may alleviate hepatic insulin resistance.

Beyond insulin resistance, AT-EVs regulate hepatic glucose and lipid metabolism. For instance, sEVs deficient in miR-141-3p impaired glucose uptake in AML12 cells [155], while Ad-sEVs with uncharacterized cargo promoted lipid accumulation, lipogenesis, ER stress, and inflammation in both C2C12 and AML12 cells [152].

AT-EVs contribute to MASLD pathogenesis through diverse mechanisms. These include facilitating hepatic lipid deposition via sEVs with reduced miR-29a-3p levels [157], delivering pro-steatotic signals via sEVs containing Aldo-keto-reductase 1B7 (AKR1B7) [158], and secreting sEVs carrying long noncoding RNA (lncRNA) like *long intergenic non-protein coding RNA 1705 (LINC01705)* [159] and TCONS-00039830 [160] that promoted hepatocyte lipid accumulation. Ad-sEVs were also taken up by hepatocytes and HSCs, disrupting TGF-β signaling and promoting fibrogenesis [161]. Additionally, AT-sEVs exacerbated MASH by increasing hepatic miR-103 levels, thereby inhibiting hepatocyte autophagy by decreasing phosphatase and tensin homolog and LC3-II/I [162].

Exercise confers metabolic benefits through AT-EV remodeling. Aerobic exercise altered miR-324 levels in AT-sEVs; upon delivery to the liver, this miRNA enhanced insulin sensitivity by suppressing ROCK1 [163]. This EV-mediated adipose-to-liver signaling axis may represent a novel mechanism for the protective effects of exercise in MASLD.

In contrast to most EVs derived from white adipose tissue (WAT), EVs derived from BAT, which are associated with thermogenesis and energy expenditure [164], mainly showed beneficial effects on liver metabolism. Interestingly, following intravenous injection, BAT-sEVs preferentially accumulated in the liver [57]. Among the top 20 proteins upregulated in these sEVs, most were components of mitochondria involved in catabolism [57]. Functionally, BAT-secreted sEVs containing miR-132-3p inhibited hepatic lipogenesis by suppressing Srebf1 [165], while BAT-EVs carrying miR-378a-3p stimulated hepatic gluconeogenesis by inhibiting *p110α* to prevent hypoglycemia during cold adaptation [166]. Additionally, BAT-derived sEVs alleviated metabolic syndrome in obese mice by reducing hepatic inflammation and lipid deposition [57]. Beyond BAT, EVs derived from adipose stem and progenitor cells carrying miR-144-3p and miR-486a-3p promoted hepatocyte proliferation by suppressing Txnip [167], suggesting broader roles for AT-EVs in liver regeneration.

Adipose depot-specific EV functions were demonstrated by the adipocyte-specific Dicer knockout (ADicerKO) mouse model, in which adipocyte-specific Dicer deletion depleted circulating miRNAs. Circulating miRNAs were restored after BAT transplantation but not after WAT transplantation [168]. In this study, BAT-sEVs carrying miR-99b suppressed hepatic fibroblast growth factor 21 (FGF21) expression, improved glucose tolerance, and reduced insulin resistance [168]. WAT transplantation, however, failed to alter serum or hepatic FGF21 levels, underscoring depot-specific functional differences in adipose-derived EV miRNAs and their distinct impacts on systemic metabolism.

#### 4.2.3. The Impacts of SkM-Derived EVs on the Liver Metabolism and Pathology of MASLD/MASH

Current evidence regarding the impact of SkM-derived EVs on the liver remains inconsistent, with both beneficial [169,170] and detrimental effects having been reported [171]. Supporting a beneficial role in MASH, remote limb ischemic conditioning (RIC) promoted the muscle-to-liver transfer of EVs carrying miR-181d-5p, which was delivered to hepatocytes and suppressed the nuclear receptor NR4A3, thereby improving hepatic metabolism, reducing hepatocyte apoptosis, and attenuating steatohepatitis [169]. Similarly, exercise-induced plasma and SkM-derived EV miRNAs, particularly miR-133a and miR-133b, were associated with improved glucose tolerance, insulin sensitivity, and decreased triglyceride levels in the plasma of sedentary mice [170]. In contrast, overtraining-induced lactate accumulation in SkM promoted the release of sEVs selectively packaged with F-box protein 2 (FBXO2), termed “lactate bodies”, which, upon delivery to the liver, promoted hepatocyte apoptosis through myeloid cell leukemia sequence 1 (MCL1) degradation and BAX/BAK pathway activation, subsequently triggering HSC-mediated liver fibrosis [171]. This detrimental signaling can be interrupted by inhibiting lactate production in muscle or by salidroside treatment. Although exercise exerts many beneficial functions crucial for health, the potential adverse effects of tissue crosstalk, particularly under excessive exercise conditions, warrant further investigation [172].

**Figure 4:**
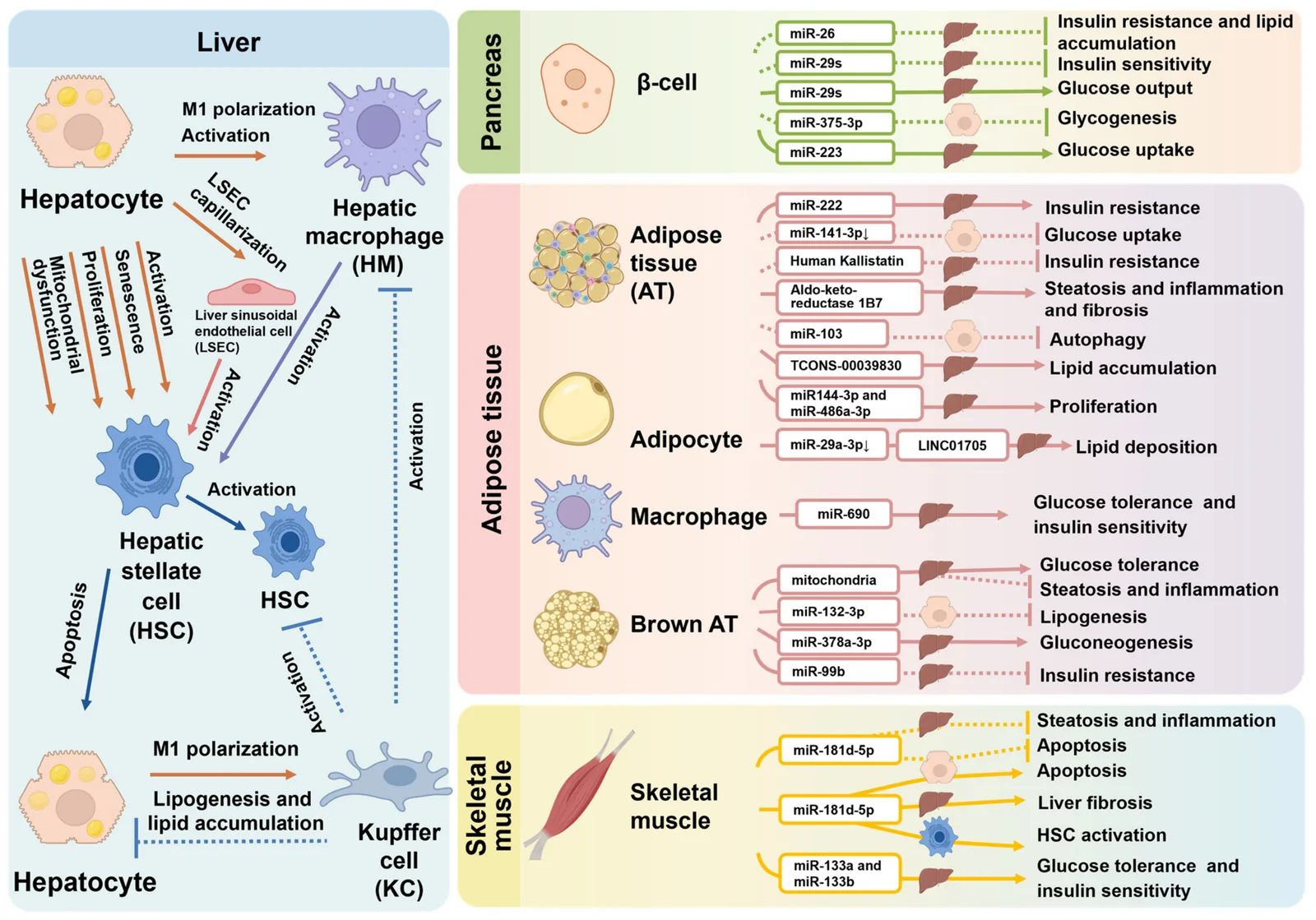
Extracellular vesicle-mediated local and systemic communication in hepatic metabolism, local environment, and pathology of MASLD and MASH. The (**left**) panel depicts the local EV-mediated crosstalk within the liver, involving hepatocyte, hepatic stellate cell (HSC), hepatic macrophage (HM), Kupffer cell (KC), and liver sinusoidal endothelial cell (LSEC). For example, EVs derived from lipotoxic hepatocytes, when delivered to HSCs, promote HSC activation, senescence, proliferation, and mitochondrial dysfunction. The (**right**) panel summarizes the systemic EV signals originating from pancreatic β-cell, adipose tissue, adipocyte, adipose tissue macrophage, brown adipose tissue, and skeletal muscle, which modulate hepatic glucose and lipid metabolism as well as the pathology of MASLD/MASH. The central box outlines the corresponding EV cargo. The text following the arrow or bar indicates the EV-mediated effects on target cells or organs. For example, pancreatic β-cell-derived EVs containing miR-26a could inhibit liver insulin resistance and lipid accumulation. In the schematic, a solid “→” indicates promotion, while a dashed “˧” denotes inhibition. The lines representing EV effects are color-coded according to the corresponding parental cells/tissues. Figures were created with BioRender (https://biorender.com/).

In summary, EV-mediated local communication primarily contributes to MASLD progression through the activation of HSC and HM, whereas EV signals originating from pancreatic islets, WAT, and SkM converge on the liver to reshape glucose and lipid metabolism as well as insulin sensitivity. Notably, BAT-derived EVs predominantly confer metabolic benefits. Figure 4 summarizes the local and systemic mechanisms of MASLD and MASH-associated EVs. Table 4 lists EV cargoes and their corresponding parental cells, recipient cells, and mechanisms.

## 5. EV-Mediated Local and Systemic Communication in the Skeletal Muscle Insulin Resistance and Sarcopenia

### 5.1. EV-Mediated Local Communication Within the SkM

Skeletal muscle (SkM) is a stable tissue with minimal cell turnover [173]; however, it possesses the capacity to regenerate after injury by various stimuli (e.g., trauma, chronic wasting disorders, and metabolic diseases [174]). Muscle satellite cells, the primary stem cell population in SkM, play a central role in muscle regeneration [175]: upon activation induced by injury or growth signals, quiescent satellite cells activate and undergo symmetric divisions to expand the satellite cell pool or asymmetric divisions to produce self-renewing stem cells and myogenic progenitors. These progenitors proliferate and differentiate into myoblasts, which subsequently undergo cell–cell fusion to form multinucleated myotubes, which either coalesce to generate de novo myofibers or fuse with damaged remnants of existing myofibers to restore muscle structure and function [176]. The divisions of satellite cells are critical for ensuring both adequate progenitor supply and the long-term maintenance of the stem cell reservoir [177].

EV-mediated intercellular communication has emerged as a key modulator of SkM biology, contributing not only to SkM homeostasis, exercise adaptation, and metabolic signal transduction under physiological conditions, but also to muscle repair and regeneration under pathological conditions [178]. Although SkM regeneration is observed in metabolic diseases like diabetes [179], most mechanistic insights are from non-metabolic models [180], such as injury [181] and hereditary muscular dystrophy [182]. Sarcopenia, a prevalent muscle complication of diabetes and obesity [183], shares pathological features with other muscle-wasting conditions, such as impaired regenerative capacity [184], and its development involves local hyperlipidaemia and insulin resistance induced by metabolic disorders [185]. Accordingly, this section focuses on EV-mediated mechanisms in sarcopenia.

These EV-mediated signals can be either pathogenic or protective, depending on their cellular origin. sEVs derived from atrophic muscle fibers and carrying miR-690 were shown to inhibit satellite cell differentiation by targeting myocyte enhancer factor 2 [46]. sEVs derived from insulin-resistant SkM, enriched in cholesterol and miRNAs, promoted adipocyte lipid storage, enhanced muscle lipid uptake, and induced insulin resistance and atrophy in muscle cells [113]. Similarly, sEVs from lipotoxically stressed muscle cells propagated lipotoxicity by intercellular lipid transfer [186]. Conversely, satellite cell-derived sEVs attenuated atrophy and fibrosis by activating PAX7 through suppression of the TGF-B1/SMAD3 pathway [187]. These findings suggest that therapeutic strategies for sarcopenia should aim to reduce pathogenic EVs originating from atrophic and metabolically stressed muscle cells while promoting the delivery or activity of protective EVs derived from satellite cells.

### 5.2. EV-Mediated Systemic Communication in Skeletal Muscle: Insulin Resistance and Sarcopenia

#### 5.2.1. The Effects of Pancreatic β-Cell-Derived EVs on the SkM Insulin Resistance

Current evidence suggests that β-cells under metabolic stress can contribute to insulin resistance in SkM. sEVs derived from palmitate-stressed β-cells enriched with miR-204-5p were taken up by SkM cells, leading to impaired insulin signaling by suppressing *Sirtuin 1*, thereby reducing glucose uptake, and ultimately contributing to systemic insulin resistance [188]. The impact of pancreatic β-cell-derived EVs on SkM remains largely unexplored and needs further exploration.

#### 5.2.2. The Effects of Adipose Tissue-Derived EVs on the SkM Insulin Resistance and Sarcopenia

In the obese state, AT-EVs induced insulin resistance in muscle cells [152], and several implicated EV miRNAs inhibited peroxisome proliferator-activated receptors, such as miR-27a-containing Ad-sEVs [32], miR-155-containing ATM-derived sEVs [39], and miR-29a [189]. The latter two miRNAs also promoted insulin resistance in the liver and in AT [39,189]. Similarly, AT- sEVs containing miR-4472 promoted insulin resistance and reduced glucose uptake in myocytes by suppressing the myocyte enhancer factor 2D/GLUT4 signaling axis [190]. ATM-derived sEVs containing miR-210-3p impaired insulin sensitivity in SkM cells by silencing GLUT4 [101]. Other factors, such as circadian disruption, may also induce the secretion of miR-22-3p-enriched sEVs by adipocytes, thereby promoting muscle insulin resistance [191].

Sarcopenia is characterized by an age-related increase in both intermuscular and intramuscular fat deposition and fibrosis, with dysfunctional crosstalk between adipose cells and myocytes playing a significant role in its pathogenesis [192]. sEVs derived from aged perimuscular AT and carrying let-7d-3p reduced the number of muscular stem and progenitor cells by inhibiting HMGA2, thereby promoting the development of aging-associated muscular atrophy [193]. However, other studies suggest that AT-EVs may ameliorate age-related muscle atrophy. AT-sEVs delivered miR-146a-5p to SkM cells, which inhibited IGF-1R to improve SkM wasting [40]. These AT-sEVs were also shown to inhibit FBX32/FOXO3 signaling signaling, thereby reducing mitochondrial autophagy and enhancing muscle fiber repair [41]. This discrepancy may reflect compensatory regulatory mechanisms of AT in response to muscle wasting. Moreover, evidence suggests that Ad-sEVs containing lncRNA-DGAT2 may regulate the adipogenic differentiation of muscle satellite cells [194]. Further studies are needed to clarify the regulatory role of EVs in adipose-muscle crosstalk.

#### 5.2.3. The Effects of Liver-Derived EVs on Sarcopenia

Sarcopenia represents a frequent complication in individuals diagnosed with MASLD [195]. In MASLD-associated sarcopenia, hepatocyte-derived miR-582-5p-enriched sEVs mediated liver-to-muscle communication, promoting muscle atrophy by activating atrogenic pathways [196]. Similarly, in a cirrhosis model induced by CCl_4_, injured hepatocytes released mtDNA-enriched EVs, which triggered SkM inflammation via the cGAS-STING pathway [197]. Blockade of the pathogenic EV secretion or its downstream signaling may represent a promising therapeutic strategy for preventing liver disease-associated muscle wasting.

In summary, EV-mediated local communication within SkM reflects the interplay between stressed myofibers and satellite cell-driven repair, whereas systemic EV signaling primarily targets insulin resistance. Moreover, AT and liver-derived EVs bidirectionally modulate SkM wasting. Table 5 summarizes EV-mediated crosstalk in SkM insulin resistance and sarcopenia, detailing cargo, parental cells, recipient cells, and mechanisms.

## 6. Conclusions and Discussion

In conclusion, EVs derived from metabolic organs, by facilitating intercellular and inter-organ communication, contribute to alterations in the local environment and systemic homeostasis, thereby influencing disease progression through their regulatory functions. In the early stages of metabolic disease, EVs mediate adaptive or compensatory responses, such as promoting pro-survival signaling and enhancing stress resilience in recipient cells. As disease progresses, beneficial EV cargo is depleted and replaced by pathogenic signals that exacerbate metabolic disease progression.

**Figure 5:**
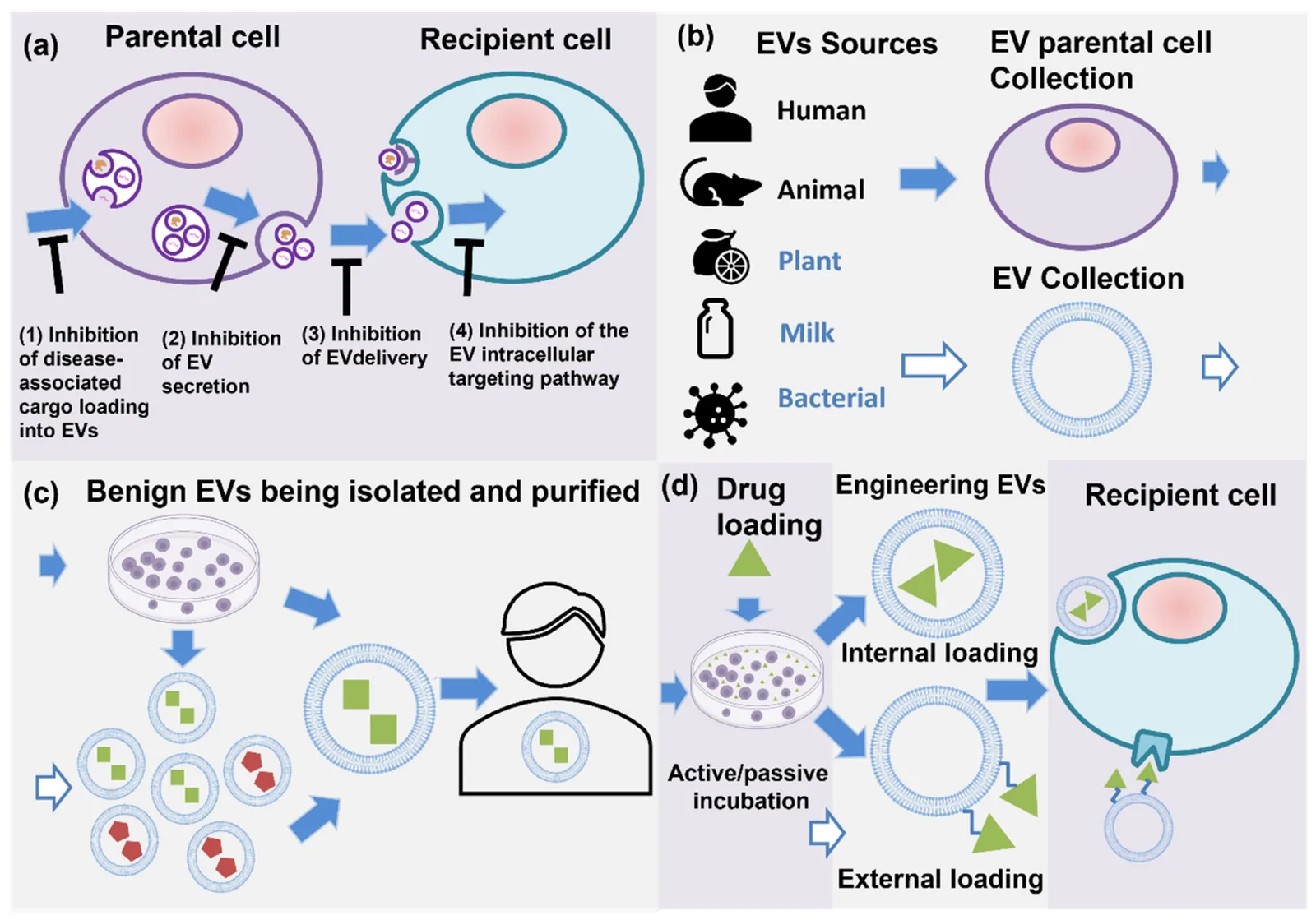
Schematic illustration of extracellular vesicle-based therapeutic strategies. (**a**) The onset and progression of diseases can be inhibited by: (1) inhibiting the loading of disease-associated cargo into EVs; (2) inhibiting the secretion of disease-associated EVs; (3) blocking the delivery of disease-associated EVs to recipient cells; (4) suppressing the target signaling pathways of disease-associated EV cargo in recipient cells. (**b**) EV sources. Parental cells of EVs or EVs can be collected from humans, animals, plants, milk, and bacteria. (**c**) Isolation and purification of benign EVs. (**d**) Drug-loaded EVs. After passive or active loading, engineered EVs carrying externally loaded cargo are suitable for delivery to the cell surface, while EVs loaded internally are suitable for cytoplasmic delivery [202]. In panel (**a**), blue arrows: 4 corresponding steps; in panels (**b**–**d**), blue arrows: steps following parental cell collection; white arrows: steps following EV collection. Figures were created with BioRender (https://biorender.com/).

Given this dual role of EVs, which shifts from adaptive protectors in early disease to pathogenic drivers in advanced stages, therapeutic strategies aimed at harnessing or counteracting EV functions have gained considerable interest. Figure 5 summarizes current EV-based therapeutic strategies. One approach is to block the loading of disease-associated cargo, inhibit EV secretion and delivery to recipient cells, and prevent the subsequent activation of pathogenic signaling. For instance, melatonin supplementation reduced the number of resistin-containing Ad-sEVs, thereby preventing hepatic steatosis [198]. A second approach is to administer highly purified benign EVs that perform their original functions [199]. These EVs can be isolated from various sources, including human and animal cells, plants, milk, and bacteria, with stem cell-derived EVs receiving particular attention [200]. Third, engineered EV-mediated drug delivery employs EVs as carriers [201], using passive or active loading methods to encapsulate therapeutic proteins, nucleic acids, or small molecules [200]. Although EVs offer superior biocompatibility and low immunogenicity compared to synthetic nanoparticles, key challenges persist. These include cargo- and method-dependent loading efficiencies, which require careful optimization, as well as the poorly characterized long-term safety, biodistribution, and metabolic behavior of EV-based formulations [201].

Table 1 summarizes a panel of shared EV cargo commonly reported across two or more metabolic disease models, including miR-27a, miR-122, miR-146a-5p, miR-155, miR-690, CXCL10, RBP4, ceramides, adipokine composition, and mitochondrial components, which may represent priority targets for future therapeutic strategies. Additional clinical evidence from circulating miRNAs but not EV-miRNAs supports the potential biomarker utility of a subset of these cargoes. Specifically, miR-27a is elevated in the plasma of patients with metabolic syndrome, with positive correlations with insulin resistance, glycemia, and lipid parameters [203]; miR-122 is increased in T2DM patients with MASLD [204]. Although these findings cannot be directly interpreted as evidence for EV-associated biomarkers, they provide support for these candidates and important clinical context for subsequent EV-fraction analyses. Clinical evidence for EV-associated miRNAs is also emerging. For example, serum EV-derived miR-122 was significantly downregulated following metabolic intervention in overweight/obese patients [205]. Furthermore, miR-155 has been identified as a predictive biomarker for incident T2DM in individuals with obesity, with the expression levels measured in both circulating plasma and EV-embedded fractions [206]. Consistent with this finding, EV-associated miR-155 levels were elevated in patients with T2DM relative to healthy controls [207].

Reproducibility remains a major barrier to the clinical translation of EV biomarkers and therapeutics, driven largely by heterogeneity in pre-analytical procedures, isolation methods, and characterization steps [208]. For example, sample processing time, storage conditions, freeze–thaw cycles, and the choice of isolation method (e.g., precipitation, differential centrifugation, density gradients, or size-exclusion chromatography) directly impact EV purity, yield, and bioactivity, which in turn affect subsequent functional outcomes. These technical discrepancies introduce confounding variables that reduce reproducibility and limit cross-study comparability [209]. Although researchers are advised to follow the MISEV guidelines when reporting methods, adherence remains inconsistent across the literature. To address these reporting gaps, the EV-TRACK consortium developed the EV-METRIC, a dynamic scoring system that incentivizes transparent documentation of isolation and characterization parameters [210]. Researchers are therefore encouraged to actively engage with this system when reporting EV studies.

## 7. Limitations and Future Prospects

Several limitations remain. (1) Due to the defined scope of this review did not encompass EV cargo profiles in the crosstalk between metabolic organs and other vital organs or tissues, which include the brain, heart, lungs, kidneys, gut, bone, and vascular endothelium. Similarly, the roles of plasma-derived or blood cell-derived EVs [211] in metabolic diseases were also not extensively reviewed. (2) The mechanisms governing the selective packaging of cargo into EVs, their release from donor cells, and their subsequent uptake by recipient cells were not comprehensively addressed in this review. (3) Most evidence is derived from cellular models and pre-clinical animal studies, which may not be directly generalizable to human populations.

We propose several directions for future research. (1) Novel methods for enriching distinct EV subtypes are needed. Priority should be given to integrating multi-omic approaches to characterize disease-specific EV cargo [212] and identify stage-specific biomarkers for early diagnosis. (2) Recent work has identified sorting motifs (EXOmotifs and CELLmotifs) that direct miRNAs into sEVs or retain them in cells [213]; whether similar “accepting motifs” exist in recipient cells is unknown. These motifs may act like sender and recipient addresses in a logistics system and may dictate cell-specific EV uptake and cargo fate.

## Figures and Tables

**Table 1:** Shared extracellular vesicle cargoes across metabolic disorders and their mechanisms of action.

Shared Cargo	Disease Model	Donor	Recipient	Molecular Target	Functions	Ref.
miR-27a	Obesity	Adipocyte	C2C12 cell	PPARγ	→ Insulin resistance	[32]
miR-27a	MASLD	Hepatocyte	HSC and hepatic Kupffer cell	PINK1	→ HSC activation, liver steatohepatitis, fibrosis, and lipidosis and mitochondrial dysfunction	[33]
miR-122	Obesity	Adipose tissue	ADSC and adipocyte	VDR and SREBF1	→ Adipogenesis	[34]
miR-122	MASLD	Hepatocyte	Adipocyte	*Pgc1α*	→ Lipogenesis and lipid accumulation ┤ Mitochondrial biogenesis and lipid oxidation	[35]
miR-122	MASH	Hepatocyte	Hepatocyte and hepatic macrophage	Inflammatory signals	→ M1 macrophage activation → Inflammatory cytokines	[36]
miR-155	Obesity and early-stage diabetes	Adipose tissue macrophage	β-cell	*Mafb*	┤ Insulin secretion → β-cell proliferation	[37]
miR-155	Obesity	Adipocyte	BMDM	SOCS1-mediated JAK/STAT signaling pathway	→ M1 macrophage phenotype	[38]
miR-155	Obesity	Adipose tissue macrophage	Muscle, liver, adipose tissue	PPARγ	→ Systemic insulin resistance → Glucose intolerance	[39]
miR-146a-5p	Skeletal muscle atrophy	Skeletal muscle	Skeletal muscle	IGF-1R or FBX32/FOXO3 signaling pathway	┤ Myofiber atrophy, apoptosis, and mitochondrial autophagy	[40,41]
miR-146a-5p	Obesity	Skeletal muscle	Adipocyte	GDF5 and PPARγ	┤ Differentiation of preadipocytes and their adipogenesis ┤ Glucose and fatty acid uptake	[42]
miR-690	Obesity	Adipose tissue macrophage	Hep, adipocytes, and muscle cell	*Nadk*	→ Glucose intolerance and insulin sensitivity	[43,44]
miR-690	MASH	Hepatic Kupffer cell	Hepatocyte, HSC, BMDM	NAD+ kinase	→ Shuttling miR-690 from Kupffer cell to cells ┤ Hepatocyte lipogenesis, HSC fibrogenesis and macrophage inflammation	[45]
miR-690	Sarcopenia	Skeletal muscle	Muscle satellite cell	Myocyte enhancer factor 2	┤ Satellite cell differentiation (myogenic capacity)	[46]
CXCL10	Diabetes	β-cell	β-cell	CXCR3	→ β-cell dysfunction and recruitment of immune cells	[47]
CXCL10	MASH	Hepatocyte	Macrophage	CXCR3	→ Macrophage chemotaxis	[48]
RBP4	Obesity	Adipose tissue	Blood monocyte	TLR4/TRIF signaling pathway	→ Differentiation into an activated M1 macrophage → Insulin resistance in vivo	[49]
RBP4	MASLD	Hepatocyte	Hepatic Kupffer cell	NOX2/ROS/NF-κB pathway	→ M1-like polarisation of Kupffer cell → Lipid accumulation and inflammation in hepatocytes	[50]
Ceramide	Diabetes	β-cell	β-cell	Insulin signaling	┤ Glucose-stimulated insulin secretion response	[51]
Ceramide	MASH	Hepatocyte	Hepatic macrophage	/	→ BMDM migration → Monocyte-derived macrophages in the liver	[52]
Adipokine composition	Obesity	ADSC	ADSC	RAS signaling, ErbB signaling, and MAPK signaling pathway	→ Adipogenesis, adipose tissue browning, and glucose metabolism ┤ Liver lipid droplet accumulation	[53]
Adipokine composition	Insulin resistance	Adipose tissue	Hepatocyte	Insulin-induced Akt phosphorylation	→ Insulin resistance	[54]
Mitochondrial components	Diabetes	β-cell	Pancreatic macrophage	REG3G/EXTL3-heparan sulfate-dependent signaling cascade that blocks NF-κB and downregulates P2RX7	┤ Pancreatic islet inflammation and proinflammatory macrophage expansion	[55]
Mitochondrial components	Obesity	Brown adipocyte	macrophage	CD36 scavenger receptor	→ Lipid-associated macrophage phenotype	[56]
Mitochondrial components	Obesity	Brown adipose tissue	Liver and visceral adipose tissue	/	→ Glucose tolerance and oxygen consumption ┤ Liver fat deposition and inflammation	[57]
Mitochondrial components	Obesity	Skeletal muscle	Adipocyte and liver	Central carbon and energy metabolism pathways	→ Glucose tolerance ┤ Lipid accumulation, liver damage, and atherosclerosis	[58]

Abbreviations: MASLD: metabolic dysfunction-associated steatotic liver disease; MASH: Metabolic dysfunction-associated steatohepatitis; PINK: PTEN-induced putative kinase 1; ADSC: Adipose-derived stem cells; VDR: Vitamin D receptor; SREBF1: Sterol regulatory element binding transcription factor 1; BMDM: Bone marrow-derived macrophage; HSC: Hepatic stellate cell; GDF5: Growth and differentiation factor 5; SOCS1: Suppressor of cytokine signaling 1; JAK: Janus kinase; STAT: Signal transducer and activator of transcription; IGF-1R: Insulin-like growth factor 1 receptor; FBX32: F-box protein 32; FOXO3: Forkhead box O3; CXCL10: C-X-C motif chemokine ligand 10; CXCR3: C-X-C chemokine receptor type 3; TLR4/TRIF: Toll-like receptor 4/Toll-interleukin-1 receptor domain-containing adaptor protein inducing interferon-β; NOX2: NADPH oxidase 2; ROS: Reactive oxygen species; MAPK: p38 mitogen-activated protein kinase; REG3G: Regenerating islet-derived 3 gamma; EXTL3: Exostosin-like glycosyltransferase 3; P2RX7: Purinergic receptor P2X7. “→” indicates promotion, while “˧” denotes inhibition.

**Table 2:** EV cargo in Diabetes (T1DM/T2DM/Pancreatogenic diabetes mellitus).

Disease Model	Donor Cell/Tissue	Recipient Cell/Tissue	Major Cargo	Target	Effects	Diagnosis/Treatment	Ref
Extracellular vesicle (EV)-mediated local communication in diabetes							
T1DM ∙T cell to islet β-cell	∙CD4+ T cell from lymphoid tissue [non-obese diabetic (NOD)/ShiLtJ mice] ∙Human CD4+ T cell (anonymous donor) ∙Human Jurkat T cell	∙MIN6B1 cell ∙Rat islet cell	miR-142-3p, miR-142-5p, and miR-155	→chemokine signaling, including *Ccl2*, *Ccl7*, and *Cxcl10*	→β-cell apoptosis →recruitment of immune cells	Adeno-associated virus (AAV) producing a transcript containing multiple binding sites for miR-142-3p/-5p, miR-150, and miR-15	[61]
T1DM ∙T cell to islet β-cell	∙CD4+ T cell (lymphoid tissue of NOD/ShiLtJ and NOD.Cg-Tg mice) ∙Human CD4+ T cell	∙MIN6B1 cell ∙Mouse islet cell	non-coding RNA fragment	/	→β-cell apoptosis	/	[62]
T2DM ∙Macrophage to islet β-cell	∙Lipopolysaccharide (LPS) and IFN-γ induced M1 bone marrow-derived macrophage (BMDM) [Wild type (WT) mice] ∙Islet-resident macrophage [Diet-induced obesity (DIO) WT mice]	∙MIN6 cell ∙Mouse primary islet	miR-212-5p	┤SIRT2 and Akt/GSK-3β/β-catenin pathway	┤insulin secretion	Knockdown of miR-212-5p in M1-sEVs by antisense	[63]
T1DM ∙Islet β-cell to T cell	∙NIT-1 cell with PD-L1 overexpression Cytokines treated ∙INS-1 cell and ∙EndoC-βH1 cell	CD8+ T cell (NOD/ShiLtJ mice)	programmed death-ligand 1 (PD-L1)	→binding to programmed death protein 1	┤proliferation and cytotoxicity of murine CD8+ T cells	Plasma EV PD-L1 positively correlated with circulating C-peptide in islet autoantibody-positive (Ab+) individuals or individuals with recent-onset T1DM	[64]
Diabetes ∙Islet β-cell to islet β-cell	Cytokine-exposed MIN6 cell	Islet β-cell (WT mice)	C-X-C Motif Chemokine Ligand 10 (CXCL10)	→binding to C-X-C Motif Chemokine Receptor 3 (CXCR3)	→β-cell dysfunction and recruitment of CD8+ T cells and macrophages	Antagonist of the CXCR3 receptor	[47]
T2DM ∙Islet β-cell to monocytes /macrophage	∙Primary islet β-cell [β-cell-specific miR-29a/b/c transgenic (βTG) mice] ∙MIN6 cell (after pre-miR-29a transfec-tion)	∙LPS stimulated BMDM (WT mice) ∙Peritoneal macrophage (βTG mice)	miR-29	┤TNF-receptor-associated factor 3	→secreting proinflammatory cytokine IL-12 in BMDMs →miR-29 level in circulating white blood cells of βTG mice	A miRNA sponge blocking miR-29	[66]
T2DM ∙Islet β-cell to macrophage	Palmitic acid (PA) treated ∙mtDsRed2-labeled MIN6 cell ∙β-TC6 cell	Macrophage ∙BMDM (WT mice) ∙RAW264.7 cell ∙Pancreatic macrophage (HFD-fed *Reg3g*-/- mice)	damaged mitochondria	→REG3G/EXTL3-heparan sulfate-dependent signaling cascade that blocks NF-κB and downregulates P2RX7	┤pancreatic islet inflammation and proinflammatory macrophages’ expansion	∙*Reg3g*-overexpressing viral vector ∙P2RX7 downregulation ∙Avoid using heparin	[55]
Diabetes ∙Islet β-cell to islet β-cell	Proinflammatory cytokines treated ∙INS-1 and MIN6 cell ∙Caffeic acid phenethyl ester treated INS-1 cell ∙Mouse islet (WT mice) ∙Human islet (Euglycemic overweight or obese individual)	INS-1 cell	∙Ceramide ∙specific miRNAs	┤insulin signaling	┤glucose-stimulated insulin secretion (GSIS) response	∙Genetic knockdown of nSMase2 ∙Glucagon-like peptide-1 receptor agonist ∙Plasma EV ceramide species increased in children with T1DM	[51]
T2DM ∙Islet β-cell to islet β-cell	∙Palmitate-treated MIN6 cell ∙Palmitate-treated healthy human islet	∙Primary islet β-cell (WT mice) ∙Healthy human islet	islet amyloid polypeptide, insulin 2, amyloid precursor protein	→TGFβ/Smad3 pathway	┤GSIS response	LY2109761, a TGFβ receptor I/II inhibitor	[65]
Pancreatogenic diabetes melli-tus ∙PSC to islet β-cell	TGF-β1-treated pancreatic stellate cell line (PSC)	∙MIN6 cell ∙Primary islet β-cell (WT mice)	miR-140-3p and miR-143-3p	┤B-cell lymphoma 2	→β-cell apoptosis ┤GSIS response	miR-140-3p and miR-143-3p inhibitors	[67]
T2DM ∙PSC to islet β-cell	∙Primary PSC cultured in hypoxia (Balb/c mice) ∙In vivo, activated intra-islet PSC (Microsphere treated Balb/c mice)	∙Primary islet β-cell (Balb/c mice) ∙In vivo islet (Microsphere treated Balb/c mice)	miR-23a-3p	/	→β-cell apoptosis ┤diameters of insulin-positive cells	Therapy targeting PSC activation	[68]
EV-mediated systemic communication in diabetes							
Obesity ∙Adipocyte to islet β-cell	Adipocyte from epididymal white adipose tissue (eWAT) (DIO WT mice)	∙Primary islet β-cell (WT mice) ∙MIN6 cell ∙Pancreas in vivo (WT mice)	insulinotropic protein	→insulinotropic GPCR/cAMP/PKA signaling	→1st-phase GSIS response ┤glucose uptake	/	[71]
Obesity ∙ATM to islet β-cell	Adipose tissue (AT) macrophage (ATM) from eWAT (DIO WT mice)	∙Primary islet β-cell (WT mice) ∙MIN6 cell ∙Human islet (overweight patient) ∙Pancreas in vivo (DIO WT mice)	miR-155	┤MAF bZIP transcription factor B	┤insulin secretion →β-cell proliferation	Knockout of miR-155	[37]
Obesity ∙Adipocyte to islet β-cell	∙3T3-L1 adipocyte treated with TNF-α/IFN-γ/IL-1β ∙Obese AT explant	∙Human EndoC-βH3 β-cell ∙INS-1E β-cell	miR-155-5p, miR-30a-5p, miR-146	┤GSK3β and MAPK ERK1/2	→β-cell apoptosis ┤β-cell proliferation ┤insulin secretion	∙EVs from healthy 3T3-L1 adipocytes ∙Human lean adipocyte-derived EVs	[73]
Obesity ∙Adipocyte to islet β-cell	∙PA treated-3T3-L1 cell ∙AT (DIO WT mice)	MIN6 cell	miR-138-5p	┤SOX4-mediatedWnt/β-catenin pathway	┤insulin secretion →β-cell apoptosis	∙SOX4 overexpression plasmid ∙miR-138-5p inhibitor	[72]
Obesity ∙Adipocyte to islet β-cell	eWAT (DIO WT mice or *db/db* mice)	∙Primary islet β-cell (WT mice) ∙MIN6 cell ∙Pancreatic islet in vivo (WT mice)	miR-27a-5p	┤L-type Ca2+ channel subtype CaV1.2	┤insulin secretion	∙Overexpression of mouse CaV1.2 ∙Depleting visceral AT miR-27a-5p	[74]
MASLD ∙Hepatocyte (Hep) to islet β-cell	PA treated ∙primary murine Hep ∙AML12 cell	MIN6 cell	miR-126a-3p	┤insulin receptor substrate-2	→β-cell apoptosis ┤insulin secretion	The antagomir against miR-126a-3p	[77]
MASLD ∙Hep to islet macrophage, β-cell and Hep	∙PA treated primary mouse Hep ∙Lipotoxic Hep-sEVs treated pancreatic macrophage ∙PA treated Huh7 human hepatocyte	∙Pancreatic macrophage in vivo (WT mice) ∙INS-1 beta cell ∙Primary islet β-cell (WT mice) ∙Peritoneal macrophage (WT or Tlr4−/− mice) ∙Pancreas and liver in vivo (WT mice) ∙Human pancreatic islet	Fetuin-A, an adaptor ligand for saturated fatty acid (SFA)-mediated inflammation	→Toll-like receptor 4 (TLR4)	┤GSIS response →hepatic and pancreatic inflammation →hepatic insulin resistance	Deletion of TLR4 in macrophages	[78]
Obesity ∙Hep to islet β-cell	Primary Hep (DIO WT mice)	MIN6 cell	miR-7218-5p	→*Cd74*	→β-cell proliferation	/	[79]
Diabetes ∙Muscle cell to islet β-cell	Primary rat muscle-derived stem cell	High glucose/palmitic acid-treated rat insulinoma INS- 1 cell line	/	→ LC3-II/I ┤p62 ┤Akt/ mTOR signaling	┤β-cell apoptosis →β-cell autophagy and insulin secretion	Muscle stem cell-derived sEVs	[83]

Abbreviation: →: promotion; ˧: inhibition; EV: Extracellular vesicle; T1DM: Type 1 diabetes mellitus; AAV: Adeno-associated virus; NOD mice: non-obese diabetic mice; T2DM: Type 2 diabetes mellitus; LPS: Lipopolysaccharide; BMDMs: bone marrow-derived macrophages; WT: Wild type micec: C57BL/6J mice or C57BL/6B mice; DIO: Diet-induced obesity; PD-L1: Programmed death-ligand 1; CXCL10: C-X-C Motif Chemokine Ligand 10; CXCR3: C-X-C Motif Chemokine Receptor 3; βTG mice: β-cell-specific miR-29a/b/c transgenic mice; PA: Palmitic acid; AT: Adipose tissue; ATM: Adipose tissue macrophages; WAT: White adipose tissue; eWAT: epididymal white adipose tissue; PSCs: Pancreatic stellate cell line; HFD: High-Fat Diet; Akt/GSK-3β: Protein kinase B/glycogen synthase kinase 3β; Reg3g: Regenerating islet-derived 3 gamma; EXTL3: Exostosin-like glycosyltransferase 3; P2RX7: Purinergic receptor P2X7; GPCR: G-protein coupled receptors; PKA: Protein Kinase A; MAPK: Mitogen-activated protein kinase; ERK: Extracellular signal-regulated kinase; SOX4: SRY (sex determining region Y)-box 4; Hep: hepatocytes; SFA: saturated fatty acid; TLR4: Toll-like receptor 4.

**Table 3:** EV cargo in Adipose tissue remodeling/Obesity.

Disease Model	Donor Cell/Tissue	Recipient Cell/Tissue	Major Cargo	Target	Effects	Diagnosis/Treatment	Ref
Extracellular vesicle (EV)-mediated local communication in adipose tissue remodeling and obesity							
Healthy ∙AT to ADSC	Adipose tissue (AT) explant [Sprague–Dawley (SD) rat]	Adipose tis-sue-derived stem cell (ADSC) [Inguinal WAT (iWAT) of SD rat]	miR-450a-5p	┤WISP2 (also known as CCN5)	→adipogenesis	miR-450a-5p overex-pression	[87]
Obesity ∙AT to ADSC and adipocyte	AT (WT mice)	∙ADSC cell line ∙3T3-L1 cell	miR-122	┤VDR →SREBF1	→adipogenesis	miR-122 inhibition	[34]
Healthy ∙ADSC to ADSC	Primary human adipose-derived stem cell (HASC) during white adipogenic differentiation	∙Primary HASC (human adipose-derived stem cell) ∙In vivo (BALB/c mice)	adipokine composition	→Leptin, FABP4, and PPARγ	→adipogenesis, lipid droplet accumulation, AT regeneration	/	[53]
Obesity ∙ADSC to ADSC	Primary HASC during beige adipogenic differentiation	∙Primary HASC ∙In vivo (DIO WT mice)	adipokine composition, miR-193b, miR-328, and miR-378a	→RAS signaling, ERBB signaling, and MAPK signaling pathway	→adipogenesis, AT browning, and glucose metabolism ┤liver lipid droplet accumulation	/	[53]
Aneurysmatic aortic disease ∙Adipocyte to monocyte	In vitro differentiated adipocyte or ex vivo AT-explant (Patient with aneurysmatic aortic disease)	Primary monocyte from healthy human	adiponectin and retinol-binding protein 4 (RBP4)	/	→differentiation into macrophages with an adipose tissue macrophage (ATM) phenotype	/	[92]
Obesity ∙AT to monocyte/macrophage	Visceral AT (DIO mice or ob/ob mice)	∙Blood monocyte ∙Bone marrow-derived macrophage (BMDM) (WT mice) ∙In vivo (WT mice)	RBP4	→TLR4/TRIF pathway	→differentiation into an activated M1 macrophage →insulin resistance in vivo	TLR4 knockout	[49]
Insulin resistant ∙Adipocyte to macrophage	High glucose and insulin cultured 3T3-L1 adipocyte	∙BMDM (WT mice) ∙RAW 264.7 macrophage	Sonic Hedgehog	→PTCH/PI3K signalling pathway	→M1 macrophage polarization	Inhibitors of PTCH and PI3K	[93]
Obesity ∙Adipocyte to macrophage	Adipocyte from eWAT (DIO WT mice)	BMDM (WT mice)	miR-155	┤SOCS1 regulates the JAK/STAT signaling pathway	→M1 macrophage phenotype	Inhibition of miR-155	[38]
Obesity ∙APC to macrophage	Adipose progenitor cell (APC) (middle-aged DIO mice)	∙ATM (In vivo) ∙BMDM (WT mice)	miR-145-5p↓	→L-selectin and NF-κB signaling pathway	→M1 macrophage phenotype	Liposomes delivering miR-145-5p mimics to ATM	[94]
Obesity ∙Adipocyte to macrophage	Adipocyte from visceral AT (VAT) (DIO WT mice)	BMDM (WT mice)	miR-34a	┤Krüppel-like factor 4	┤M2 macrophage polarization →insulin resistance	Adipose-selective or adipocyte-specific miR-34a knockout	[95]
Obesity ∙AT to macrophage and adipocyte	eWAT (DIO WT mice treated with melatonin)	∙Primary peritoneal macrophage (WT mice) ∙Adipocyte (DIO mice)	α-ketoglutarate	→TET1, TET2, and TET3 ┤STAT3/NF-κB signal pathway	→M2 macrophage and DNA demethylation ┤adipose inflammation	∙Melatonin Treatment ∙α-ketoglutarate treatment	[96]
Obesity ∙ADSC to macrophage and WAT	ADSC from eWAT (WT mice)	∙Peritoneal macrophage (WT mice) ∙eWAT in vivo (DIO WT mice)	Active STAT3	→Arginase-1	→M2 macrophage polarization→Beiging in WAT and ┤WAT Inflammation	ADSC-derivedsEVs	[97]
Obesity ∙AT to macrophage	Perigonadal adipose tissue (WT mice)	∙ATM (ob/ob mice) ∙Bone marrow precursor	lipid	/	→lipid accumula-tion →differentiation into ATM phenotype	PI3K inhibitor	[98]
Healthy ∙Adipocyte to adipocyte	∙Large eWAT adipocyte and plasma (SD rat) ∙Adipocyte differentiated from rat stromal vascular cell (SVC) for 10 days (SD rat)	∙Small eWAT adipo-cyte (SD rat) ∙Adipocyte differen-tiated from rat SVC for 1 day (SD rat)	Gce1 and CD73, LD-and adi-pokine-specific transcripts, miR-16, miR-27a, miR-222, miR-146b	→lipid droplet (LD)-associated pro-teins and adipokines: FSP27, caveolin-1, GPAT-3, DGAT-2, leptin, and adiponec-tin	→lipogenesis and adipocyte hyper-trophy	/	[99]
Diabetic obesity ∙Macrophage to adipocyte	∙High glucose-induced RAW264.7 macrophage ∙Serum (DIO mice treated with streptozotocin for 3 days)	3T3-L1 adipocyte	miR-210	┤Nicotinamide Adenine Dinucleotide dehydrogenase ubiq-uinone 1 alpha sub-complex 4 (NDUFA4)	┤glucose uptake and mitochondrial CIV complex activ-ity	Inhibition of miR-210 or miR-210 knockout	[100]
Obesity ∙Macrophage to adipocyte and AT, Liver, SkM	∙BMDMs (WT mice) and RAW264.7 cells transfected with miR-210-3p mimic ∙ATM from eWAT (DIO mice)	∙3T3-L1 adipocytes ∙VAT, liver, and skeletal muscle (SkM) in vivo (WT mice)	miR-210-3p	┤GLUT4	┤glucose uptake →systemic insulin resistance	Inhibition of miR-210-3p in VAT	[101]
Healthy ∙EC to adipocyte	AT endothelial cell (EC)	Adipocyte (mouse over-expressing a mini-SOG-tagged cav1 fusion protein specifically in EC)	caveolin	/	sustaining the Cav1 level	/	[102]
Obesity ∙Adipocyte to macrophage	∙PA treated murine T37i adipocyte ∙Primary brown adipocyte (db/db or DIO mice) labeled with mitotracker	∙Murine RAW 264.7 cell ∙BMDM	damaged lipids and mitochondria	→CD36 scavenger receptor	→lipid-associated macrophages phenotype	Inhibition of CD36	[56]
Cold stress ∙Adipocyte to adipocyte and macrophage	∙Brown adipose tissue (BAT) (WT mice at 4℃)	∙Murine T37i adipocyte ∙Primary brown adipocyte (WT mice)	damaged mitochondria	┤peroxisome pro-liferator-activated receptor-γ (PPAR-γ) signaling ┤UCP1	┤thermogenic response to cold exposure	Keeping phagocytic activity of BAT-resident macrophage	[103]
Healthy ∙BAT to adipocyte	BAT (WT mice)	∙3T3-L1 cell ∙ADSC	AK029592	/	→beige adipocyte differentiation	/	[104]
Healthy ∙Adipocyte to adipocyte	Primary brown adipocyte [human mitochondrial transcription factorA(TFAM) homozygous transgenic (TgTg) mice]	Primary brown adipocyte (WT mice)	TgTg EVs: release ↑, cargo unchanged vs WT mice	/	→oxidative phosphorylation and mitochondrial activity	TFAM overexpression	[105]
Obesity ∙AT to AT	iWAT (WT mice)	BAT and WAT (DIO mice)	miR-23b	┤E74 like ETS transcription factor 4 and GLP-1R	→obesity and insulinresistance ┤thermogenesis of BAT and WAT browning	miR-23b antagomir	[106]
EV-mediated systemic communication in adipose tissue remodeling and obesity							
T2DM and obesity ∙Islet β-cell to AT and liver	∙Pancreatic β-cell (Pancreatic β-cell–specific miR-26a TG mice) ∙Min6 cell overexpressing miR-26a	∙Liver tissue, VAT, and BAT (Pancreatic β-cell–specific miR-26a TG mice) ∙Primary murine Hep (WT mice)	miR-26a	┤*Inhba* and *Dnmt3a* in the liver →*Ucp3*, *Pnpla2*, *Pgc1a*, *Pparg* and ┤*Adam17* in the WAT	┤adipocyte hypertrophy ┤insulin resistance ┤hepatic steatosis and lipid accumulation	∙Serum sEVs with reduced miR-26a as a biomarker for T2D ∙Overexpression of miR-26 in β-cells	[107]
MASLD ∙Hepatocyte (Hep) to adipocyte	Primary Hep (DIO WT mice)	Differentiated 3T3-L1 preadipocyte	let-7e-5p and miR-122	┤*Pgc1α*	→lipogenesis and lipid accumulation ┤mitochondrial biogenesis and lipid oxidation	∙Liver *Ggpps* deletion ∙Rab27A geranylgeranyl site mutation ∙Anti-let-7e-5p-EV	[35]
Early onset obesity ∙Hep to adipocyte and other tissues	Hep (DIO WT mice)	∙AT, SkM, and liver (DIO mice) ∙3T3-L1 cell, L6 myocyte	miR-3075	┤FA2H	┤insulin resistance →glucose uptake	miR-3075 mimic-containingliposomes	[109]
Chronic obesity ∙Hep to ATM and other tissues	Hep (DIO WT mice)	∙AT, SkM, and liver (lean WT mice) ∙BMDM (WT mice)	miR-434-3p	→NF-κB	→Proinflammatory ATMs accumulation in eWAT →insulin resistance →tissue inflammation	∙Depletion of hepatocyte *Rab27* or *Ybx1* ∙miR-434-3p antagomir	[109]
Obesity ∙Hep to adipocyte and other tissues	Liver tissue (miR-130a-3p overexpression mice with HFD)	∙3T3-L1 cell ∙AT, liver, lung, kidney, SkM (miR-130a-3p knock-out mice on an HFD)	miR-130a-3p	┤PHLPP2 →AKT-AS160-GLUT4 signaling pathway	┤adipogenesis and lipid droplets →glucose uptake	miR-130a-3p overexpression	[110]
MASLD ∙Hep to BAT	AML12 cell transfected with HA tag-conjugatedmouse Tm4sf5	∙Primary brown adipose cell ∙BAT (Tm4sf5 −/− mice on a high-carbohydrate diet)	TM4SF5	→phosphorylation of mTOR	→glucose uptake →BAT thermogenesis	TM4SF5 overexpression	[111]
Obesity ∙Hep to BAT	Cold-stimulated primary Hep (WT mice)	Immortalized brown adipocyte, primary brown adipocyte, and beige adipocyte	miR-293-5p	┤Ten-eleven translocation methylcytosine dioxygenase 1 (TET1)	→BAT thermogenesis ┤DIO-related metabolic dysfunctions	∙Cold exposure ∙miR-293-5p agomir	[112]
Obesity ∙SkM to adipocyte and muscle cell	Quadricep and gastrocnemius muscle (ob/ob mice)	∙3T3-L1 cell ∙C2C12 muscle cell	lipid, protein, and miRNA	→lipid metabolism-related targets ┤AKT2 protein but →insulin receptors in muscle	→lipid storage in adipocyte →altered insulin-sensitivity →atrophy in the muscle	/	[113]
Obesity ∙SkM to adipocyte and liver	SkM (exercised WT mice)	Liver and AT (DIO mice and HFD-fed *Apoe-/-* mice)	mitochondrial and fatty acid oxidation-related components	→central carbon and energy metabolism pathways	→glucose tolerance ┤lipid accumulation, liver damage, and atherosclerosis	Therapeutic delivery of exercise-induced EVs or the analogues	[58]
Healthy ∙SkM to adipocyte	SkM (mechanical loading WT mice)	∙3T3-L1 adipocyte ∙eWAT (exercise naïve WT mice)	∙muscle-specific miR-1↓ ∙serum EV miR-1↑	┤TFAP2α then→ADRβ3	→adrenergic signaling and lipolysis	/	[115]
Healthy ∙SkM to adi-pocyte	SkM (Human vastus lateralis, baseline and 45 minutes after acute resistance exercise)	AT (Human, baseline and 45 minutes after acute resistance exercise)	∙muscle-specific miR-1↑ ∙Serum sEV miR-1↑	┤caveolin 2 (CAV2) and tripartite motif containing 6 (TRIM6)	→adipose miR-1 (with no effects on Adipose pri-miR-1b) →catecholamine-induced lipolysis	∙Overexpression of miR-1 ∙Resistance exercise	[116]
Obesity ∙SkM to adipocyte	∙C2C12 cell during the proliferation stage ∙SkM (SkM-specific knockout miR-146a-5p mice)	∙3T3-L1 cell ∙AT (SkM-specific knockout miR-146a-5p mice)	miR-146a-5p	┤growth and dif-ferentiation factor 5 (GDF5) and PPARγ Signaling	┤differentiation of preadipocytes and their adipogenesis ┤glucose and fatty acid uptake	miR-146a-5p mimic	[42]

Abbreviation: →: promotion; ˧: inhibition; EV: Extracellular vesicle; AT: Adipose tissue; SD rat: Sprague–Dawley rat; iWAT: Inguinal white adipose tissue; ADSC: Adipose tissue-derived stem cell; WT: Wild type mice: C57BL/6J mice or C57BL/6B mice; WAT: White adipose tissue; BAT: Brown adipose tissue; WISP2: Wnt-inducible signaling pathway protein 2; VDR: Vitamin D3 receptor; SREBF1: Sterol regulatory element-binding transcription factor 1; HASC: Human adipose-derived stem cell; DIO: Diet-induced obesity; MAPK: Mitogen-activated protein kinase; RBP4: Retinol-binding protein 4; ATM: Adipose tissue macrophage; BMDM: Bone marrow derived macrophage; TLR4/TRIF: Toll-like receptor 4/Toll-interleukin-1 receptor domain– containing adaptor protein inducing interferon-β; SOCS1: Suppressor of cytokine signalling-1; JAK/STAT: Janus kinase/signal transducers and activators of transcription signaling; Tet: Ten-eleven translocation methylcytosine dioxygenase; STAT3: Signal transducer and activator of transcription 3; PI3K: Phosphatidylinositol 3-kinase; SVC: Stromal vascular cell; LD: Lipid dtoplet; DGAT2: Diacylglycerol O-acyltransferase 2; NDUFA4: Nicotinamide Adenine Dinucleotide dehydrogenase ubiquinone 1 alpha subcomplex 4; SkM: Skeletal muscle; VAT: Visceral adipose tissue; TFAM: mitochondrial transcription factor A; GLUT4: Glucose transporter type 4; TM4SF5: Transmembrane 4 L six family member 5; mTOR: Mammalian target of rapamycin; AKT2: Protein Kinase B Beta; CAV2: caveolin 2; TRIM6: tripartite motif containing 6; GDF5: growth and differentiation factor 5.

**Table 4:** EV cargo in MASLD/MASH.

Disease Model	Donor Cell/Tissue	Recipient Cell/Tissue	Major Cargo	Target	Effects	Diagnosis/Treatment	Ref
Extracellular vesicle (EV)-mediated local communication in the liver microenvironment and pathology of MASLD and MASH							
MASLD ∙Hepatocyte (Hep) to HSC	∙Palmitic acid (PA) treatedHuman hepatoma cell line (HepG2) ∙PA treated primary mouse Hep (PMH) (WT mice)	∙Human hepatic stellate cell (HSC) (LX2 cell) ∙Primary mouse HSC (WT mice)	miR-128-3p	┤PPAR-γ	→HSC migration, proliferation, and activation	Inhibition of miR-128-3p	[123]
MASLD /MASH ∙Hep to HSC and KC	∙PMH (Western diet in-duced MASH mice) ∙PA-treated lean mouse hep	∙HSC [lean Kupffer cell (KC)-depleted mice and WT mice] ∙KC (lean KC-depleted mice)	iron	→ROS accumulation	→HSC fibrogenic activation	∙Hep-specific *Rab27* knockout ∙Diet-switch to enhance KC-mediated EV clearance	[124]
MASH ∙Hep to HSC	∙PMH (ccl4-induced or MCD-induced liver fibrosis mice) ∙TNF-α- treated Human hepatic cell (LO2 cell)	∙Primary HSC ∙LX2 cell	mannan-binding lectin serine protease 1 (MASP1)	→p38 MAPK/ATF2 signaling	→HSC activation	*Arrb1* knockout	[125]
MASLD ∙Hep to HSC	∙PA treated LO2 cell ∙PA treated PMH	LX2 cell	miR-1297	┤PTEN →PI3K/AKT signaling pathway	→HSC activation and proliferation	/	[126]
MASLD ∙Hep to HSC and KC	∙PA treated LO2 cell ∙LO2 cell pretransfected with mi-miR27a	∙Primary HSC ∙Primary KC ∙Liver (MASLD mice)	miR-27a	┤PINK1	→HSC activation →liver steatohepatitis, fibrosis, and lipidosis ┤mitochondrial functions	sEVs withmiR27a inhibition	[33]
MASLD ∙Hep to HSC	∙Oleic acid and PA(OPA) treated LO2 cell ∙OPA-treated PMH ∙LO2 cells transfected with *Lima1*-shRNA lentivirus	∙LX2 cell ∙HFD induced MASLD mice	LIM Domain And Actin Binding 1 (LIMA1)	┤PINK1-Parkin mediated mitophagy	→HSC activation →liver fibrosis	*Lima1* knockdown	[127]
MASLD ∙Hep to HSC	OPA treated HepG2 cells	LX-2 cells and Liver in vivo (DIO WT mice)	fatty acid synthase (FASN)	→oxidative stress	→HSC activation →area of liver fibrosis	FASN inhibitor	[128]
MASLD ∙Hep to HSC and T cell	PA-treated human normal Hep	∙LX-2 cell ∙CD4 + T lymphocyte (from healthy donors’ blood mononuclear cell)	miR-107	→WNT signaling by ┤ DKK1 in HSC → IL-9 expression by ┤ FOXP1 in T cell	→HSC activation →Th9 cell differentiation	∙DKK1 and IL-9 overexpression ∙miR-107 inhibitor	[129]
MASLD ∙Hep to HSC	∙PA treated HepG2 cell ∙PMH (MASLD mice induced by western diet +CCl4 and CDAHFD)	∙LX-2 cell ∙Primary HSC	Pumilio protein 1 (PUM1)↓	→TPM4	→HSC activation	∙PUM1 overexpression ∙*Tpm4* knockdown	[130]
MASH ∙Hep to macrophage	Palmitate or plysophosphatidylcholine (LPC) treated PMH (WT mice) or Huh7 cell	Bone marrow-derived macrophage [BMDM (WT mice)]	CXCL10	→CXCR3	→macrophage chemotaxis	Mixed lineage kinase 3 inhibitor	[48]
MASH ∙Hep to macrophage	∙PA or LPC treated PMH, and Huh7 cell ∙Serum or Hep (FFC diet-fed mice) ∙MASH patients’ serum	∙BMDM ∙THP-1 cell	tumor necrosis factor-related apoptosis-inducing ligand (TRAIL)	→NF-κB pathway activation	→an inflammatory phenotype in macrophages	∙*Death receptor 5 (DR5)* deletion ∙ROCK1 inhibitor fasudil	[132]
MASH ∙Hep to macrophage	PMH transduced withadenovirus or adeno-associated virus that expressed IRE1A	∙BMDM (WT mice) ∙Liver in vivo [High fat, fructose, and cholesterol (FFC) diet induced MASH mice]	C16:0 ceramide	/	→BMDM migration →monocyte-derived macrophage in the liver	∙IRE1A inhibitor 4μ8C ∙Hepatocyte-specific *Ire1a* knockout	[52]
MASLD ∙Hep to macrophage	∙Serum [High-fat high-cholesterol diet fed SD rats] ∙PA-treated HepG2 cell	∙Hepatic macrophage activation in vivo ∙THP-1 macrophage	miR-192-5p	┤RICTOR/AKT1/FOXO1 signaling pathway	→M1 macrophage activation	miR-192-5p inhibitor	[133]
MASLD ∙Hep to macrophage	∙PA-treated Human normal hepatocytes LO2 cell ∙Plasma (MASLD patient)	THP-1 macrophage	miR-9-5p	┤transglutaminase 2 (TGM2)	→M1 macrophage activation	∙AAV-mediated silencing of miR-9-5p ∙miR-9-5p inhibitor	[134]
MASH ∙Hep to macrophage and Hep	∙miR-122 expressing Hepa1-6 cell ∙cholesterol-lipid treated HuH7 cell ∙cholesterol-treated PMH (normal BALB/c mice)	∙Hepatic non-parenchymal cell (normal BALB/c mice) / RAW264.7 cell ∙HeLa cells/ differentiated U-937 macrophage /HepG2 ∙Primary macrophage	miR-122 with matrix metalloprotease 2 (MMP2)	→Inflammatory signals	→M1 macrophage activation →inflammatory cytokines	Inhibition of MMP2	[36]
MASH ∙Hep to macrophage	PA-treated immortalized mouse hepatocyte cell line	BMDM (WT mice)	sphingosine 1-phosphate (S1P1)	→S1P1 receptor (S1P1R)	→chemotactic responses of macrophage	∙Pharmacological inhibition of Sphingosine kinases 1 and 2 ∙*S1p1r* knockout	[135]
MASH ∙Hep to monocyte	Toxic lipid mediator LPC-treated Alpha mouse liver 12 (AML12) cell line or Huh7 cell	∙Primary bone marrow monocyte ∙Human monocyte cell line THP1	active integrin β1	→integrin signaling	→monocyte adhesion and liver inflammation	anti-ITGβ1 neutralizing antibody (ITGβ1Ab)	[136]
MASLD ∙Hep to KC	PA-treated LO2 cell	Human KC	retinol-binding protein 4 (RBP4)	→NOX2/ROS/NF-κB pathway	→M1-like polarisation of KC →lipid accumulation and inflammation in Hep	Disruption of *Rbp4*	[50]
MASLD ∙Hep to macrophage and KC	∙PA treated PMH (standard chow diet-fed mice) ∙PMH (HFD-fed mice) ∙Blood (methionine–choline-deficient diet-fed mice and HFD-fed mice) ∙Huh7 cell line ∙ Plasma (MASLD patient)	∙Peritoneal macrophage (thioglycollate-elicited mice) ∙Primary KC (standard chow diet-fed mice) ∙Liver in vivo (TLR4fl/fl or TLR4∆Mye mice) ∙Human macrophage (buffy coats from healthy donor)	palmitic (C16:0) and stearic (C18:0) saturated fatty acid	→Toll-like receptor-4 (TLR4)	→inflammatory responses in macrophages →liver inflammation and insulin resistance	Pharmacological inhibition or deletion of *Tlr4*	[138]
MASLD ∙Hep to LSEC	∙OPA-treated HepG2 cell ∙Serum of MASLD patient	∙Liver sinusoidal endothelial cell (LSEC) ∙In vivo (HFD-induced MASLD mice)	O-linked N-acetylglucosamine glycosyltransferase (OGT)	→O-GlcNAc glycosylation of the hepatocyte nuclear factor 1-alpha (HNF1α)	→ LSEC capillarization	O-GlcNAcylation inhibitor Benzyl-α-GalNAc	[139]
Liver fibrosis ∙HSC to Hep	TGFβ1-activated Rat hepatic stellate cell HSC-T6 cell	Rat HepBRL-3A	miR-99a-5p	┤BMPR2	┤Hep proliferation →Hep apoptosis	miR-99a-5p inhibition	[140]
MASH ∙KC to Hep, HSC, and macrophage	KC [AIN76A NASH Western Diet-induced MASH mice and normal chow diet (NCD)-fed WT mice]	∙PMH (NCD WT mice) ∙HSC (NCD WT mice) ∙Lipopolysaccharide (LPS)-treated BMDM	miR-690	┤NAD+ kinase	shuttling miR-690 to cells ┤HSC fibrogenesis ┤macrophage inflammation ┤Hep lipogenesis	∙miR-690 mimic ∙Nicotinamide mononucleotide (NMN) treatment	[45]
Liver fibrosis ∙HSC to HSC	∙CCN2-green fluorescent protein (GFP)–transfectedprimary mouse HSC or human LX-2 cell	∙Activated or quiescentprimary mouse HSC ∙LX-2 cell	connective tissue growth factor (CCN2)	/	→HSC fibrogenic signaling	GW4869: sEVs inhibitor	[141]
Liver fibrosis ∙Macrophage to HSC	Lipopolysaccharide-treated THP-1 macrophage	LX2 cell	miR-103-3p	┤Krüppel-like factor 4 (KLF4)	→HSC proliferation and activation	∙miR-103-3p inhibition ∙miR-103-3p in serum sEVs as a biomarker	[142]
Liver fibrosis ∙LSEC to HSC	Primary LSEC (Aldosterone-induced liver fibrosis SD rat)	Primary HSC (SD rat)	miR-342-5p↓	→TGF-BETA pathway	→HSC activation and liver fibrosis	∙miR-342-5p mimics ∙si-Atg5 AAV	[143]
EV-mediated systemic communication in the liver microenvironment and pathology of MASLD and MASH							
Obesity ∙Islet β-cell to Hep	∙Free fatty acids (FFAs) treated primary pancreatic islet ∙miR-29s-overexpressing MIN 6 cell or miR-29s-depleted MIN 6 cell	∙Primary Hep (WT mice) ∙Liver in vivo (mice with pancreatic β-cell overexpressed miR-29s)	miR-29s	┤p85α, the regulatory subunit of PI3K	┤liver insulin sensitivity →liver glucose output	Disruption of miR-29s	[147]
T2DM ∙Islet β-cell to Hep	∙High glucose treated MIN-6 cell ∙Primary islet β-cells (DIO WT mice)	Hepatic cell line Hep1-6	miR-375-3p	┤*Rbpj* ┤AKT/GSK signaling pathway	┤hepatic glycogenesis	∙miR-375-3p inhibition ∙*Rbpj* overexpression	[149]
Obesity ∙Islet β-cell to the liver and SkM	∙Primary islet β-cell (ob/ob mice) ∙High glucose-treated β-cell line MIN-6	Skeletal muscle (SkM) and liver tissue	miR-223	→GLUT4 expression	→liver and SkM glucose uptake	/	[150]
Aneurysmaticaortic disease ∙AT to Hep and muscle cell	Human subcutaneous and omental adipose tissue (AT)-explant	∙HepG2 cell ∙C2C12 myoblast	insulin-resistant adipokines	┤insulin-induced AKT phosphorylation	→insulin resistance	/	[54]
Obesity ∙AT to Hep and SkM	Gonadal WAT (gWAT) (DIO mice)	∙Hepa 1-6 cell ∙Liver and SkM of DIO mice	miR-222	┤insulin receptor substrate 1 (IRS1) and phospho-AKT level	→insulin resistance ┤glucose tolerance	Surgically removing the gWAT	[154]
Obesity ∙AT to Hep	Visceral AT (ob/ob mice and DIO mice)	AML12 cell	miR-141-3p↓	┤phospho-AKT level	→insulin resistance ┤glucose uptake	∙GW4869 ∙Overexpression of miR-141-3p	[155]
Obesity ∙AT to Hep	∙eWAT (DIO mice injected with Ad.HKS adenovirus) ∙3T3L1 cell transfected with Ad. HKS	∙Liver in vivo (DIO mice) ∙PA-treated AML12 cell	human Kallistatin (HKS)	→phosphorylation of AKT	┤liver insulin resistance	High expression of HKS in epididymal adipose tissue	[156]
Obesity ∙macrophage to Hep, adipocyte, and muscle cell	IL4/IL13-induced BMDM macrophage (WT mice)	∙Liver, AT, and SkM (DIO mice) ∙Primary Hep, 3T3-L1 adipocyte, and L6 myocyte	miR-690	┤*Nadk*	→glucose tolerance ┤insulin resistance	∙miR-690 Overexpression ∙*Nadk* suppression	[43]
Obesity ∙Macrophage to Hep, muscle cell, and adipocyte	∙ATM (rosiglitazone-treated DIO mice) ∙Rosiglitazone-treated BMDM	∙L6 myotube, 3T3-L1 adipocyte, and primary mouse and human Hep) ∙Pancreatic islet (DIO mice)	miR-690	┤*Nadk*	→glucose tolerance and insulin sensitivity ┤AT inflammation →GSIS	∙miR-690 mimic ∙Rosiglitazone treatment	[44]
Bisphenol S exposure ∙Adipocyte to Hep	Bisphenol S exposure-treated 3T3-L1 adipocyte	AML12 cell	miR-29a-3p↓	→*Col4a1*	→hepatic lipid deposition	miR-29a-3p overexpression	[157]
MASH ∙AT to Hep	∙eWAT (DIO mice with recombinant lentiviral vector of *Akr1b7*) ∙adipocyte pre-treated with tunicamycin	∙PMH ∙Liver (DIO mice, methionine-choline-deficient diet (MCD)-induced NASH mice)	Aldo-keto-reductase 1B7	→NF-κB pathway	→triglyceride and FFA levels →hepatic steatosis, inflammation, and fibrosis	Depletion of *Akr1b7*	[158]
Diabetes ∙Adipocyte to Hep	High glucose-stimulated human adipocyte cell	HepG2 cell	*LINC01705*	┤miR-552-3p and →LXR	→lipid deposition	Knockdown of LINC01705	[159]
MASLD+ obstructive sleep apnoea ∙AT to Hep	eWAT (HFD-fed SD rat simulated by chronic intermittent hypoxia)	Oleate and palmitate treated PMH (chow diet fed SD rat)	TCONS-00039830	┤miR-455-3p and →SMAD2	→lipid deposition	miR-455-3p overexpression	[160]
MASH ∙AT to Hep	∙Abdominal AT and plasma (DIO mice injected with miR-103 antagonist) ∙3T3-L1 cell	∙Liver in vivo (mice model unknown) ∙OPA-treated AML-12 cell	miR-103	┤phosphatase and tensin homologand LC3-II/I→ p-AMPKa, p-mTOR, and p62	┤hepatocyte autophagy ┤number of autophagosome →MASH pathology	Inhibition of miR-103	[162]
MASLD ∙AT to Hep	∙Plasma (MASLD patient with aerobic exercise) ∙AT (DIO mice with exercise)	∙HepG2 cell (treated with OPA and insulin) ∙PMH	miR-324	┤*ROCK1*	┤liver fat deposition →hepatic insulin sensitivity	miR-324-5p overexpression	[163]
Obesity ∙BAT to Hep	Interscapular BAT (healthy WT mice)	∙Liver and VAT (DIO mice) ∙AML12 cell	mitochondrial components	/	→glucose tolerance ┤expression of inflammatory genes ┤liver fat deposition →oxygen consumption	BAT-sEVs isolated from young healthy mice	[57]
Cold adaptation ∙BAT to Hep	∙Differentiated primary brown adipocyte (treated with norepinephrine) ∙BAT (cold stimuli treated WT mice)	∙PMH ∙AML12 cell	miR-132-3p	┤*Srebf1* expression	┤hepatic lipogenesis	Cold stimulation	[165]
Cold adaptation ∙BAT to Hep	∙Interscapular BAT (WT mice) ∙Primary brown adipocytes transduced with a lentivirus vector encoding either GFP	∙PMH (WT mice) ∙Liver in Vivo (WT mice)	miR-378a-3p	┤*p110α*	→hepatic gluconeogenesis	Cold stimulation	[166]
Adipose tissue regeneration ∙AT to Hep	Serum or iWAT (adipose stem- and progenitor cell) [Tamoxifen-inducible adipocyte-specific insulin receptor knockout (iFIRKO) mice]	∙Hepa 1–6 cell ∙PMH (WT mice) ∙Liver in vivo (iFIRKO mice)	miR144-3p and miR-486a-3p	┤*Txnip* expression	→hepatocyte proliferation	EV-miR144-3p and miR-486a-3p as regenerative medicine	[167]
Lipodystrophy ∙BAT to Hep	∙BAT (Lox-control mice) ∙ADicerKO sEVs reconstituted with miR-99b	∙Liver (ADicerKO mice) ∙AML-12 cell	miR-99b	┤fibroblast growth factor 21 expression (FGF-21)	→glucose tolerance ┤Insulin resistance	/	[168]
MASH ∙SkM to Hep	∙Hindlimb muscle distal to the tourniquet and plasma [remote limb ischemic conditioning (RIC)-treated MASH mice] ∙Plasma (human volunteers undergoing RIC)	∙PA-treated PMH ∙Liver in vivo (CDAHFD and HFHC diet-induced MASH mice) ∙PA-treated primary human Hep	miR-181d-5p	┤nuclear receptor 4A3 (NR4A3)	┤Hep cell death, steatosis, and inflammatory cytokine production ┤mouse MASH phenotype	AAV8 encoding miR-181d-5p sponge	[169]
Healthy liver fibrosis with overtraining ∙SkM to Hep	∙SkM (healthy overtrained mice with AAV-*Fbxo2*-zsGreen intramuscularly injection, CCl4-treated and MCD-fed WT mice) ∙C2C12 myotube treated with lactate	∙Hepa1-6 ∙PMH (WT mice) ∙LX-2 cell	F-box protein 2 (FBXO2)	┤MCL1 and the activated BAX/BAK pathway	→Hep apoptosis →HSC activation and liver fibrosis	∙*Ldha* deletion ∙Salidroside treatment	[171]
Sedentary ∙SkM to Hep	Muscle (high-intensity interval training WT mice)	∙PMH ∙Liver in vivo (Sedentary WT mice)	miR-133a and miR-133b	┤transcriptionfactor *forkhead box O1 (FoxO1)*	→glucose tolerance and insulin sensitivity	Exercise-induced sEVs carrying miRNA	[170]

Abbreviation: →: promotion; ˧: inhibition; EV: Extracellular vesicle; MASLD: Metabolic dysfunction-associated steatotic liver disease; MASH: Metabolic dysfunction-associated steatohepatitis;PA: Palmitic acid; HepG2 cell: Human hepatoma cell line; LX2 cell: Human hepatic stellate cell; LO2 cell: Human hepatic cell; AML12 cell: Alpha mouse liver 12 cell line; PMH: Primary mouse hepatocyte; HSC: Hepatic stellate cell; WT: Wild type mice:C57BL/6J mice or C57BL/6B mice; KC: Kupffer cell; MCD: Methionine-choline-deficient diet; ROS: Reactive oxygen species; MASP1: Mannan-binding lectin serine protease 1; MAPK: Mitogen-activated protein kinase: ATF2: Activating transcription factor 2: ARRB1: Arrestin beta 1; PTEN: Phosphatase and tensin homolog deleted on chromosome ten; PI3K/AKT: Phosphoinositide 3-kinase/Protein kinase B; PINK1: PTEN-induced kinase 1; FASN: Fatty acid synthase; PUM1: Pumilio protein 1; TPM4: Tropomyosin 4; OPA: Oleic acid and palmitic acid; LIMA1: LIM Domain And Actin Binding 1; LPC: Phosphatidylcholine; CXCL10: C-X-C Motif Chemokine Ligand 10; CXCR3: Cell-Surface (C-X-C motif) receptor 3; FFC: High fat: fructose: and cholesterol; DIO: Diet-induced obesity; TRAIL: Tumor necrosis factor-related apoptosis-inducing ligand; DR5: Death receptor 5; ROCK1: Rho-associated coiled-coil containing protein kinase 1; IRE1A: also called ERN1: Endoplasmic reticulum to nucleus signaling 1; Akt1/FoxO1: Threonine-protein kinase/the Forkhead box O; TGM2: Transglutaminase 2; MMP2: Matrix metalloprotease 2; S1P1: Sphingosine 1-phosphate; RBP4: Retinol-binding protein 4; NOX2: NADPH oxidase 2; ROS: Reactive oxygen species; LSECs: Liver sinusoidal endothelial cells; OGT: O-linked N-acetylglucosamine glycosyltransferase; HNF1α: Hepatocyte nuclear factor 1-alpha; BMPR2: Bone morphogenetic protein receptor type 2; LPS: Lipopolysaccharide; CCN2: Connective tissue growth factor; FFAs: Free fatty acids; Rbpj: Recombination signal binding protein for the immunoglobulin kappa J region; SkM: Skeletal muscle; GLUT4: Glucose transporter type 4; IRS1: Insulin receptor substrate 1; Nadk: NAD(+) kinase; Col4a1: Collagen type IV alpha 1 chain; FFA: Free fatty acid; Srebf1: Sterol regulatory element binding transcription factor 1; iFIRKO: Tamoxifen-inducible adipocyte-specific insulin receptor knockout; ADicerKO: Adipocyte-specific Dicer knockout; FGF-21: Fibroblast growth factor 21 expression; RIC: Remote limb ischemic conditioning; CDAHFD: Choline-deficient: L-amino acid-defined: high-fat diet; HFHC: High-fat and high-cholesterol diet; NR4A3: Nuclear receptor 4A3; FBXO2: F-box protein 2; MCL1: Myeloid cell leukemia sequence 1; BAX/BAK: Bcl-2-associated X Protein/Bcl-2 homologous antagonist/killer; FoxO1: Forkhead box O1.

**Table 5:** EV cargo in Skeletal muscle Insulin Resistance and Sarcopenia.

Disease Model	Donor Cell/Tissue	Recipient Cell/Tissue	Major Cargo	Target	Effects	Diagnosis/Treatment	Ref
Extracellular vesicle (EV)-mediated local communication in skeletal muscle insulin resistance and sarcopenia							
Sarcopenia ∙Skeletal muscle (SkM) to muscle satellite cell (SC)	∙Starvation-induced atrophic myotube ∙Tibialis anterior (TA) muscle (aged WT mice)	∙C2C12 myoblast during differentiation ∙Satellite cell in TA muscle (atrophic myotubes-sEVs-treated WT mice)	miR-690	┤myocyte enhancer factor 2	┤satellite cell differentiation (myogenic capacity)	Specific silencing of miR-690 in the muscle via recombinant Adeno-associated virus (AAV)-miR-690-sponge	[46]
Obesity ∙SkM to muscle cell	Quadricep and gastrocnemius muscle (leptin-deficient ob/ob mice)	C2C12 muscle cell	altered lipid, protein, and miRNA	→nuclear genes involved in lipid metabolism	→lipid uptake and fatty acid oxidation →insulin resistance and atrophy	/	[113]
Insulin resistance ∙SkM to muscle cell	∙Quadriceps muscle (palm oil-fed WT mice) ∙PA treated C2C12 cell ∙Oleate-treated C2C12 cell	∙Differentiated C2C12 myotube ∙Liver, spleen, and lungs in vivo (WT mice)	palmitate	┤*Myod1* and *Myog* →*Ccnd1*	→myoblast proliferation	/	[186]
SkM atrophy ∙SC to SkM	SkM satellite cell	Denervated SkM [Sprague Dawley (SD) rat]	unstated carg	┤TGF-B1/SMAD3 pathway→Pax7	┤muscle atrophy and fibrosis	SkM satellite cells- sEVs-based therapy	[187]
EV-mediated systemic communication in skeletal muscle insulin resistance and sarcopenia							
Insulin resistance ∙Islet β-cell to muscle cell)	PA-treated MIN6 β-cell	C2C12 myoblast cell	miR-204-5p	┤*Sirtuin 1*	┤glucose consumption and uptake →insulin resistance	miR-204-5p inhibitor	[188]
Obesity ∙Adipocyte to muscle cell	PA-treated 3T3-L1 adipocyte	C2C12 cell	miR-27a	┤PPARγ	→insulin resistance	∙Knockdown of miR-27a ∙Rosiglitazone treatment	[32]
Obesity ∙AT to SkM	∙Adipose tissue macrophage (ATM) [Visceral adipose tissue (VAT) of HFD-fed WT mice]	∙SkM, liver, and AT in vivo (WT mice) ∙L6 muscle cell	miR-155	┤PPARγ	→insulin resistance →glucose intolerance	∙miR-155 knockout ∙ATM-EVs from lean mice	[39]
Obesity ∙AT to muscle cell	∙ATM (VAT of HFD-fed WT mice)	∙L6 muscle cell ∙3T3-L1 adipocyte ∙Primary mouse hepatocyte (PMH)	miR-29a	┤PPAR-δ	→insulin resistance ┤glucose uptake of muscle cell and adipocyte →glucose output of PMH	∙Knockdown of miR-29a ∙PPAR-δ agonist GW501516	[189]
Obesity ∙Adipocyte to SkM	∙3T3-L1 cell transfected with miR-4472 ∙Serum (Obesity patient)	∙C2C12 cell ∙SkM (DIO WT mice)	miR-4472	┤MEF2D/GLUT4	┤glucose uptake →insulin resistance	miR-4472 inhibitor	[190]
Circadian disorder ∙Adipocyte to muscle cell	Pre-adipocyte fibroblast 3T3-Ll transfected with adenovirus-sh-Bmal1	C2C12 myoblast cell	miR-22-3p	┤activation of AKT signaling	→insulin resistance	∙miR-22-3p inhibitor ∙sEVs secretion inhibitor GW4869	[191]
Sarcopenia ∙AT to muscle cell	Perimuscular AT (Aged WT mice)	Primary myoblast cell (Young WT mice)	let-7d-3p	┤HMGA2	→adipogenic differentiation	Nuclear factor-kappa B inhibitor	[193]
SkM atrophy ∙AT to muscle cell	WAT (adipose-specific miR-146a-5p knockout mice)	C2C12 myoblast cell	miR-146a-5p↓	┤suppression of IGF-1R	→muscle atrophy	miR-146a-5p mimics	[40]
SkM atrophy ∙AT to SkM	WAT (miR-146a-5p flox/flox mice)	∙Cardiotoxins treated C2C12 cell ∙TA muscle (adipose-specific miR-146a-5p knockout mice)	miR-146a-5p	┤FBX32/FOXO3 signaling	→SkM wasting ┤myofiber atrophy, apoptosis, and mitochondrial autophagy	miR-146a-5p mimics	[41]
Healthy ∙Adipocyte to SC	Bovine primary pre-adipocyte (Subcutaneous AT of Yanbian yellow cattle calves)	Primary muscle SC (longissimus dorsi muscle tissue of Yanbian yellow cattle calve)	lncRNA-DGAT2	┤bta-miR-2455 → DGAT2	→adipogenic differentiation	/	[194]
MASLD ∙Hepatocyte (Hep) to muscle cell	∙Liver (High-fat diet-induced MASLD mice) ∙PA-treated HepG2 cell	Differentiated C2C12 myotube	miR-582-5p	→atrogenic pathways include FBXO32 and TRIM63	→muscle atrophy	MnO_2_ mesoporous PD hydrogel	[196]
Sarcopenia in the cirrhosis model ∙Hep to muscle cell	Liver tissue (cirrhosis mouse model)	RAW264.7 cell or C2C12-derived myotube	mtDNA	→cGAS-STING pathway	→SkM inflammation	∙GW4869 treatment ∙*Rab27a* knockdown	[197]

Abbreviation: →; promotion; ˧: inhibition; EV: Extracellular vesicle; SkM: Skeletal muscle; TA: Tibialis anterior; SD rat: Sprague–Dawley rat; WT: Wild type mice: C57BL/6J mice or C57BL/6B mice; SC: Satellite cell; AAV:Adeno-associated virus; DIO: Diet-induced obesity; AT: Adipose tissue; VAT: Visceral adipose tissue; PMH: Primary mouse hepatocytes; WAT: White adipose tissue; IR: Insulin resistance; TA: Tibialis anterior; MEF2D: Myocyte enhancer factor 2D; GLUT4: Glucose transporter type 4; HMGA2: High-mobility group AT-Hook2; IGF-1R: Insulin-like growth factor 1 receptor; Fbx32/FoxO3: F-box protein 32/Forkhead Box O3; DGAT2: Diacylglycerol acyltransferase 2; MASLD: Metabolically active steatotic liver disease; cGAS-STING: cyclic GMP-AMP synthase-stimulator of interferon genes.

## Data Availability

No new data were created or analyzed in this study. Data sharing is not applicable to this article.
